# Metformin ameliorates the severity of experimental Alport syndrome

**DOI:** 10.1038/s41598-021-86109-1

**Published:** 2021-03-29

**Authors:** Kohei Omachi, Shota Kaseda, Tsubasa Yokota, Misato Kamura, Keisuke Teramoto, Jun Kuwazuru, Haruka Kojima, Hirofumi Nohara, Kosuke Koyama, Sumio Ohtsuki, Shogo Misumi, Toru Takeo, Naomi Nakagata, Jian-Dong Li, Tsuyoshi Shuto, Mary Ann Suico, Jeffrey H. Miner, Hirofumi Kai

**Affiliations:** 1 Department of Molecular Medicine, Graduate School of Pharmaceutical Sciences, Kumamoto University, 5-1 Oe-honmachi, Chuo-ku, Kumamoto, 862-0973 Japan; 2 Program for Leading Graduate School “HIGO (Health Life Science: Interdisciplinary and Glocal Oriented) Program”, Graduate School of Pharmaceutical Sciences, Kumamoto University, 5-1 Oe-honmachi, Chuo-ku, Kumamoto, 862-0973 Japan; 3 Department of Pharmaceutical Microbiology, Graduate School of Pharmaceutical Sciences, Kumamoto University, 5-1 Oe-honmachi, Chuo-ku, Kumamoto, 862-0973 Japan; 4 Department of Environmental and Molecular Health Sciences, Graduate School of Pharmaceutical Sciences, Kumamoto University, 5-1 Oe-honmachi, Chuo-ku, Kumamoto, 862-0973 Japan; 5 Division of Reproductive Engineering, Center for Animal Resources and Development (CARD), Kumamoto University, 2-2-1 Honjo, Chuo-ku, Kumamoto, 860-0811 Japan; 6 Center for Inflammation, Immunity and Infection, Institute for Biomedical Sciences, Georgia State University, Petit Science Center, 100 Piedmont Ave SE, Atlanta, GA 30303 USA; 7 Division of Nephrology, Washington University School of Medicine, 4523 Clayton Ave., St. Louis, MO 63110 USA

**Keywords:** Kidney, Chronic kidney disease, Nephrology, Glomerular diseases, Alport syndrome

## Abstract

Metformin is widely used for the treatment of type 2 diabetes, and increasing numbers of studies have shown that metformin also ameliorates tumor progression, inflammatory disease, and fibrosis. However, the ability of metformin to improve non-diabetic glomerular disease and chronic kidney disease (CKD) has not been explored. To investigate the effect of metformin on non-diabetic glomerular disease, we used a mouse model of Alport syndrome (*Col4a5* G5X) which were treated with metformin or losartan, used as a control treatment. We also investigated the effect of metformin on adriamycin-induced glomerulosclerosis model. Pathological and biochemical analysis showed that metformin or losartan suppressed proteinuria, renal inflammation, fibrosis, and glomerular injury and extended the lifespan in Alport syndrome mice. Transcriptome analysis showed that metformin and losartan influenced molecular pathways-related to metabolism and inflammation. Metformin altered multiple genes including metabolic genes not affected by losartan. Metformin also suppressed proteinuria and glomerular injury in the adriamycin-induced glomerulosclerosis mouse model. Our results showed that metformin ameliorates the glomerular sclerosis and CKD phenotype in non-diabetic chronic glomerular diseases. Metformin may have therapeutic potential for not only diabetic nephropathy but also non-diabetic glomerular disease including Alport syndrome.

## Introduction

Chronic kidney disease (CKD) leading to end stage kidney disease (ESKD) is one of the major global health problems with high incidence, poor prognosis, and high medical cost. There are many causes of CKD, including hypertension, diabetes, and genetic mutation; regardless of the cause, CKD commonly manifests proteinuria, renal inflammation, and fibrosis^1–4^. Alport syndrome is a hereditary glomerular disease caused by mutation in the *COL4A3, A4,* or *A5* gene encoding type IV collagen α3–5 (α3–5(IV)) chains, which are components of the glomerular basement membrane^5,6^. Patients with Alport syndrome present with chronic glomerular dysfunction, renal inflammation, and fibrosis which are the hallmarks of CKD that progress to ESKD^7^.

Metformin, widely used for treatment of type 2 diabetes^8^, inhibits mitochondrial respiratory chain complex 1 and transiently decreases mitochondrial energy production^9^. This energetic stress increases intracellular AMP level, leading to AMP-activated protein kinase (AMPK) activation^10^. AMPK is a metabolic sensor and has several beneficial effects on intracellular homeostasis, such as regulating glycolysis and lipid metabolism^11^. As shown by various lines of evidence, AMPK activation is important for the beneficial effect of metformin on type 2 diabetes treatment^12–14^. In addition to diabetic-associated diseases, several studies have reported that metformin ameliorates tumor progression, inflammatory disease, and tissue fibrosis^15,16^. Considering its relatively low cost and safety, metformin is useful not only for diabetes-related diseases but also for non-diabetic diseases.

Previous studies showed that metformin ameliorates renal inflammation and fibrosis in experimental mouse models, including unilateral ureteral obstruction (UUO)^14^, cisplatin-induced tubular injury^17^, and ischemia–reperfusion (I/R) injury^18,19^, all of which are classified as acute kidney injury. However, whether metformin has effects on non-diabetic CKD, especially on glomerular diseases, remains unknown. Here, we investigated the effect of metformin on chronic glomerular disease using a mouse model of Alport syndrome (*Col4a5* G5X mutant mice)^20^. This Alport syndrome mouse model spontaneously shows progressive glomerular disease and CKD phenotypes, including renal inflammation and fibrosis. Using this model, we demonstrate that metformin has a protective effect on non-diabetic and chronic glomerular disease.

## Results

### Metformin protects against progressive renal dysfunction in *Col4a5* G5X Alport syndrome mice

**Figure 1:**
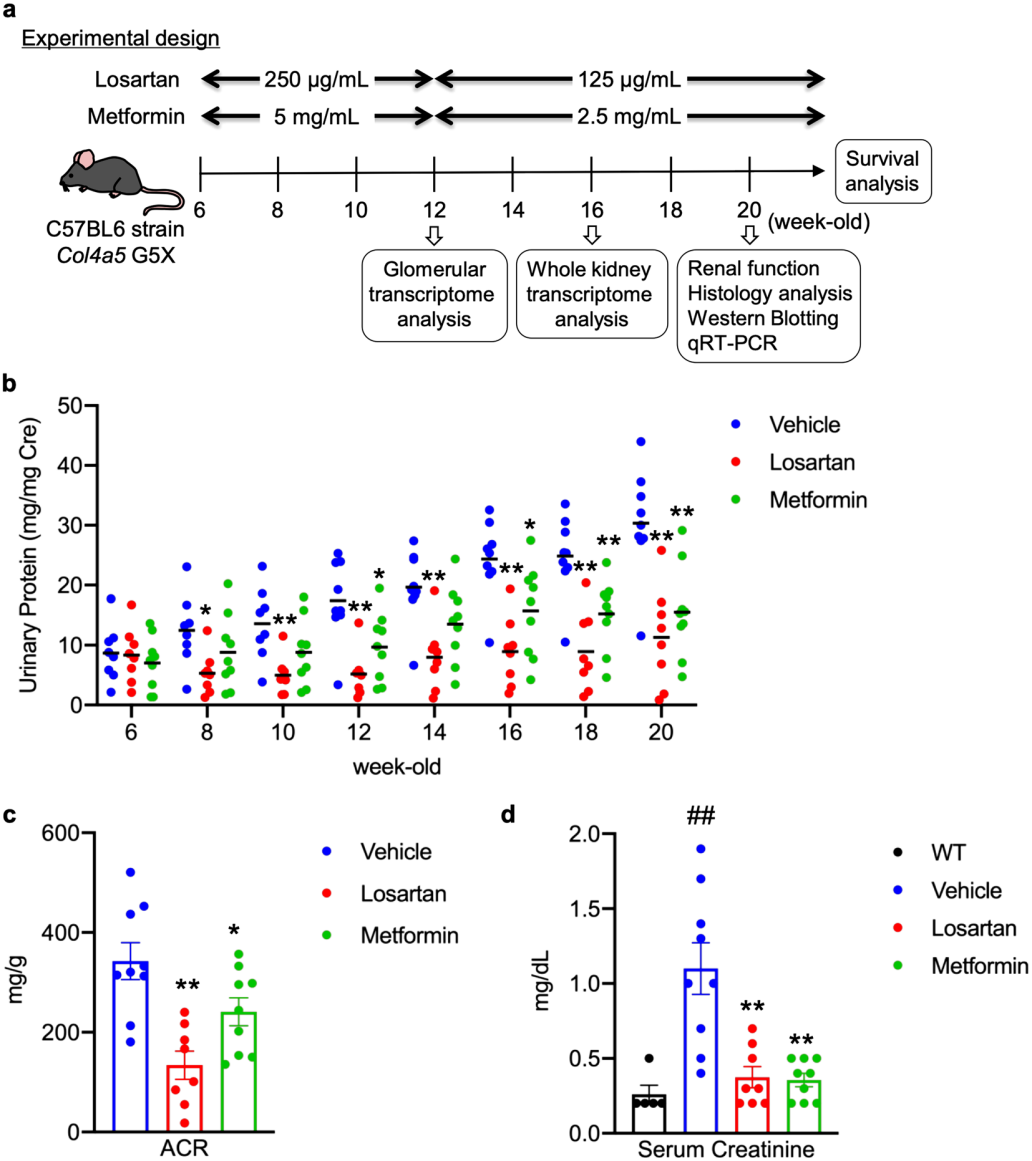
Metformin protects against progressive renal dysfunction in *Col4a5* G5X Alport syndrome mice. (**a**) The experimental design of studies performed on C57BL/6 *Col4a5* G5X Alport syndrome mice is shown. The image was drawn by S.K. (**b**) Proteinuria score was calculated based on urinary protein and creatinine concentrations. Proteinuria was reduced in losartan or metformin-treated C57BL/6 *Col4a5* G5X Alport syndrome mice. (**c**) Creatinine-normalized urinary albumin concentration was reduced in losartan- or metformin-treated C57BL/6 *Col4a5* G5X Alport syndrome mice. (**d**) The elevation of serum creatinine level in late stage C57BL/6 *Col4a5* G5X Alport syndrome mice was suppressed by losartan or metformin treatment. Data are expressed as the means ± S.E. in WT (n = 5), vehicle-, losartan- or metformin-treated C57BL/6 *Col4a5* G5X Alport syndrome mice (n = 8–9 per group). *P* values were assessed by Dunnett’s test (^##^*P* < 0.01 vs WT. *P < 0.05, **P < 0.01 vs vehicle).

To compare the effect on glomerular disease, we treated *Col4a5* G5X Alport syndrome mice with metformin or losartan, an angiotensin II receptor blocker, as a positive control therapy. Losartan is protective against various glomerular diseases, and is currently prescribed for patients with CKD^21–23^. During treatment starting at 6 weeks old to 20 weeks old (Fig. 1a), we monitored proteinuria once every two weeks and assessed albuminuria and serum creatinine at 12 and 20 weeks old, respectively. Metformin or losartan significantly reduced proteinuria, albuminuria, and serum creatinine level (Fig. 1b–d). Although losartan was more suppressive on proteinuria than metformin, metformin also significantly reduced proteinuria. The reduction of serum creatinine was similar with both treatments. Because metformin is an anti-diabetic agent with reported increased risk of lactic acidosis^24^, we evaluated its effect on body weight, water intake, urine volume, blood glucose and serum lactate levels in *Col4a5* G5X Alport syndrome mice. Metformin or losartan did not induce changes in these parameters (Supplementary Figs. S1, S2).

### Metformin suppresses renal inflammation and fibrosis in *Col4a5* G5X Alport syndrome mice

**Figure 2:**
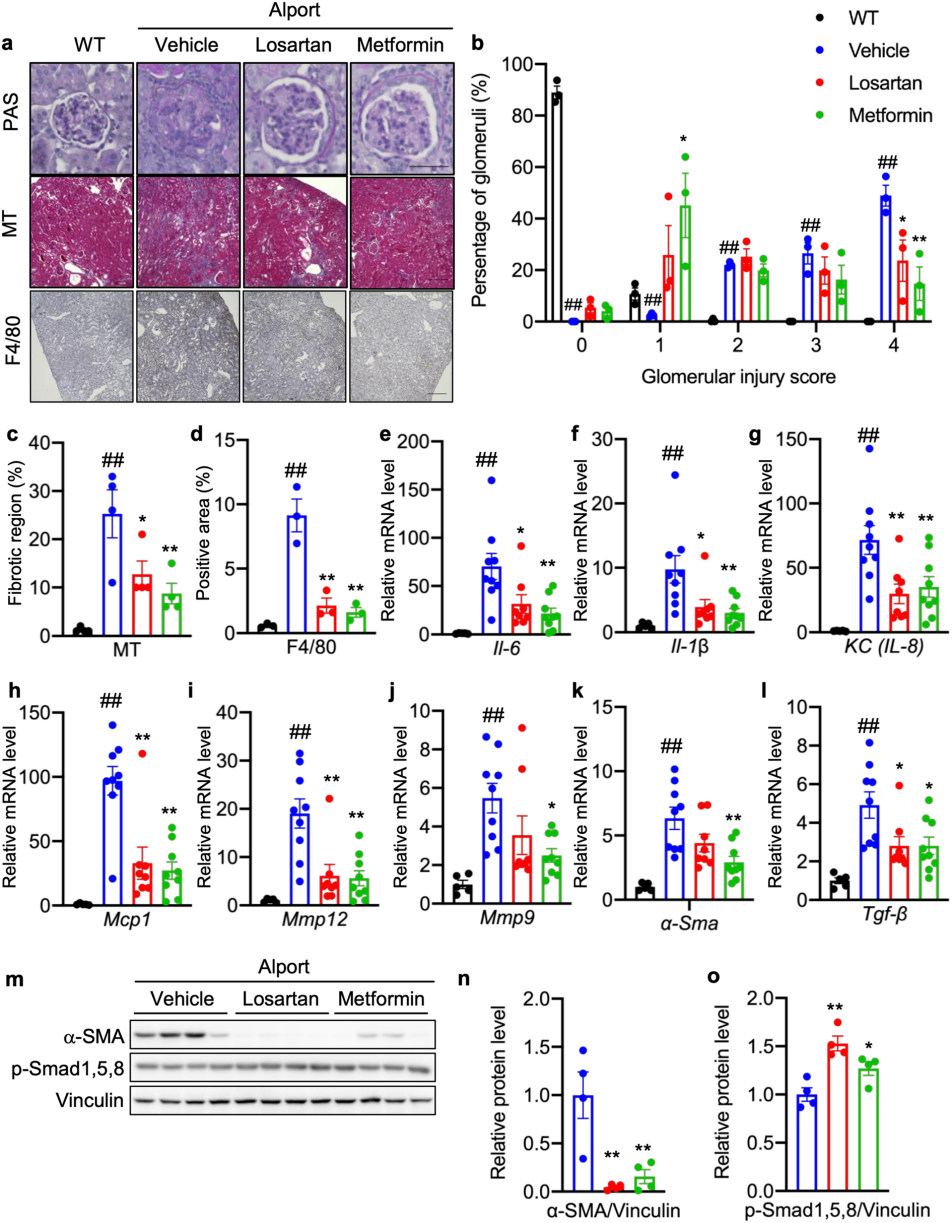
Metformin suppresses renal inflammation and fibrosis in *Col4a5* G5X Alport syndrome mice. (**a**) Staining of renal sections of 20-week-old mice by PAS, Masson-Trichrome, and F4/80 immunohistochemistry (IHC) indicated renal inflammation was ameliorated in losartan- or metformin-treated C57BL/6 *Col4a5* G5X Alport syndrome mice. Scale bars, PAS 50 μm; MT and F4/80 200 μm. (**b**) Glomerular injury scores were evaluated based on the PAS-stained sections. The severity of glomerulosclerosis was decreased in losartan- or metformin-treated C57BL/6 *Col4a5* G5X Alport syndrome mice. (**c**) Tubulointerstitial fibrosis scores were evaluated based on the MT-stained sections. The fibrotic region was reduced in losartan- or metformin-treated C57BL/6 *Col4a5* G5X Alport syndrome mice. (**d**) F4/80-positive region was evaluated based on the F4/80 IHC section. Both losartan and metformin suppressed the infiltration of macrophages. (**e**–**l**) Total RNA was isolated from renal tissues of 20-week-old mice, and subjected to quantitative RT-PCR. The data were normalized to Gapdh. Data are expressed as the means ± S.E. in WT (n = 5), vehicle-, losartan-, and metformin-treated C57BL/6 *Col4a5* G5X Alport syndrome mice (n = 8–9 per group). *P* values were assessed by Dunnett’s test (^##^*P* < 0.01 vs WT. *P < 0.05, **P < 0.01 vs vehicle). (**m**) Whole kidney lysates were analyzed by immunoblotting. The full-length blots are presented in Supplementary Fig. S11. (**n**,**o**) The relative amount of proteins was quantified. Bars indicate the mean ± S.E. (n = 4 per group). *P* values were assessed by Dunnett’s test (*P < 0.05, **P < 0.01 vs vehicle).

We evaluated the effect of metformin on common characteristics of CKD—glomerular injury, renal inflammation, and fibrosis. Severe glomerular injury (score 4), assessed by PAS staining, was detected in more glomeruli (50%) in untreated *Col4a5* G5X Alport syndrome mice (vehicle group) compared respectively with 25% and 15% in losartan- or metformin-treated Alport syndrome mice (Fig. 2a,b). Conversely, 45% of glomeruli exhibited mild injury (score 1) in metformin-treated *Col4a5* G5X Alport syndrome mice. These data indicate that losartan and metformin suppressed severe glomerular injury in Alport syndrome. Renal fibrosis, a final common pathway of CKD, was significantly reduced in the kidneys of losartan- or metformin-treated *Col4a5* G5X Alport syndrome mice compared with control (Fig. 2a,c; MT). Renal inflammation was evaluated by calculating the area positive for F4/80, a macrophage marker. Metformin or losartan suppressed macrophage infiltration (Fig. 2a,d) in kidney tissue. Correlating with this pathological analysis, the expression levels of pro-inflammatory genes *Il-6, Il-1β,* *KC/Il-8,* and *Mcp1* were down-regulated by losartan and metformin (Fig. 2e–h). Consistent with reduced fibrosis, the expression levels of fibrotic genes *Mmp9/12, *α*-Sma,* and *Tgf-β* were lower in kidney tissues of metformin- or losartan-treated *Col4a5* G5X Alport syndrome mice (Fig. 2i–l). Metformin or losartan reduced the level of α-SMA and increased the level of anti-fibrotic molecules phosphorylated Smad 1,5,8 (p-Smad 1,5,8) in kidney lysates (Fig. 2m–o), suggesting that metformin or losartan suppressed renal fibrosis.

### Metformin regulates various intracellular signaling pathways in kidney of *Col4a5* G5X Alport syndrome mice

**Figure 3:**
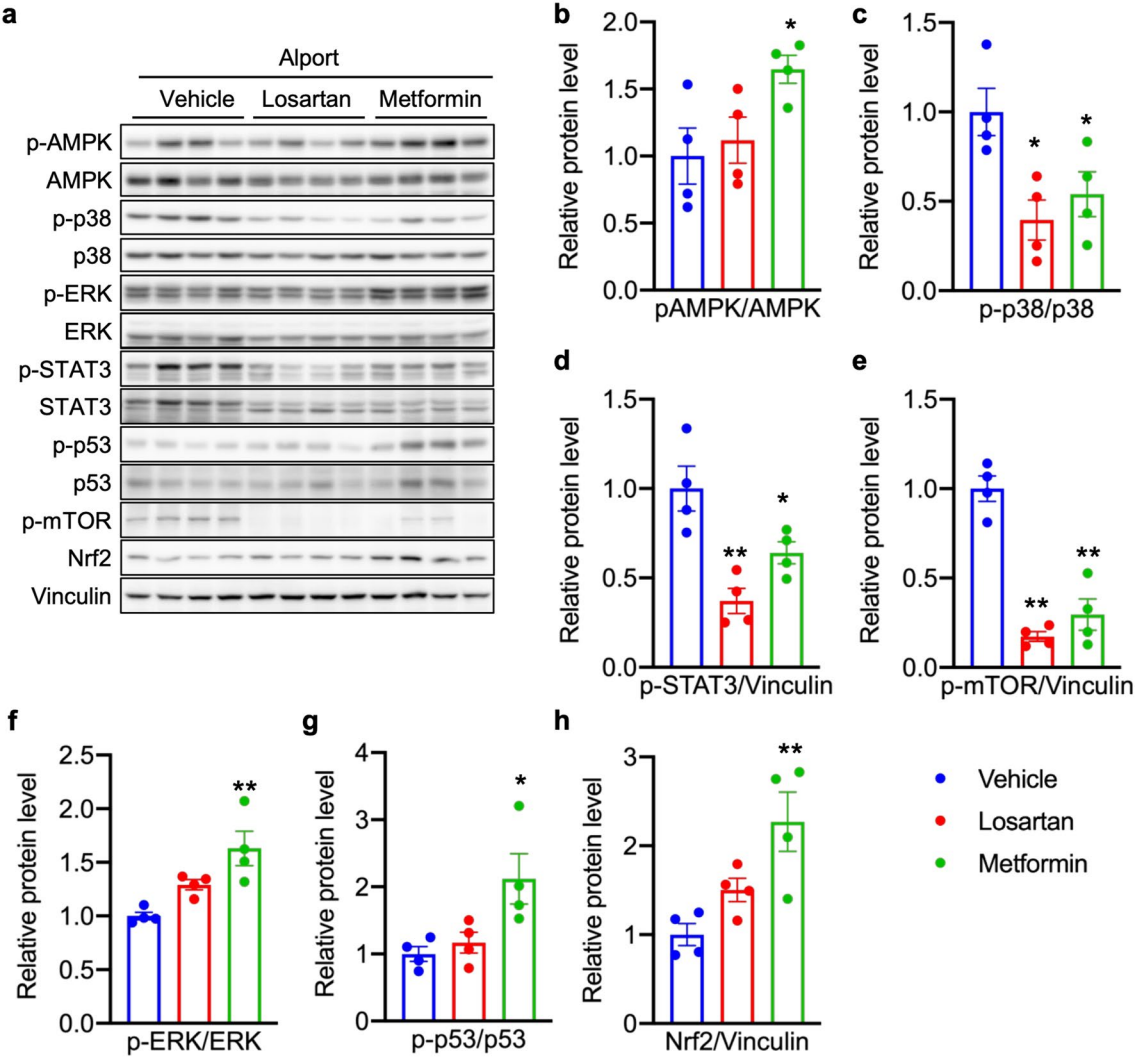
Metformin regulates various intracellular signaling pathways in kidney of *Col4a5* G5X Alport syndrome mice. (**a**) Whole kidney lysates were analyzed by immunoblotting. The full-length blots are presented in Supplementary Fig. S12. (**b**–**h**) The relative amount of proteins was quantified. Losartan or metformin decreased the level of (**c**) phospho-p38, (**d**) phospho-STAT3, (**e**) phospho-mTOR. Only metformin increased the level of (**a**) phospho-AMPK, (**f**) phospho-ERK, (**g**) phospho-p53 and (**h**) Nrf2. Data are expressed as the means ± S.E. (n = 4 per group). *P* values were assessed by Dunnett’s test (*P < 0.05, **P < 0.01 vs vehicle).

Metformin activates AMPK, which regulates intracellular homeostasis via multiple intracellular signaling pathways^11,25^. To investigate the effect of metformin on intracellular signaling, we evaluated the expression of AMPK-associated proteins in kidney lysates of metformin- or losartan-treated *Col4a5* G5X Alport syndrome mice. Metformin, but not losartan, increased phosphorylated AMPK (p-AMPK; Fig. 3a,b). Metformin and losartan each reduced the levels of phosphorylated p38 (p-p38), STAT3 (p-STAT3) and m-TOR (p-mTOR) (Fig. 3a,c–e). Only metformin increased the levels of phosphorylated ERK (p-ERK), p53 (p-p53) and NRF2 (Fig. 3a,f–h). These data indicate that in addition to the pathways that metformin and losartan similarly affect, metformin can influence other signaling pathways that are not affected by losartan.

### Whole kidney transcriptome analysis reveals the molecular effects of metformin on CKD in *Col4a5* G5X Alport syndrome mice

**Figure 4:**
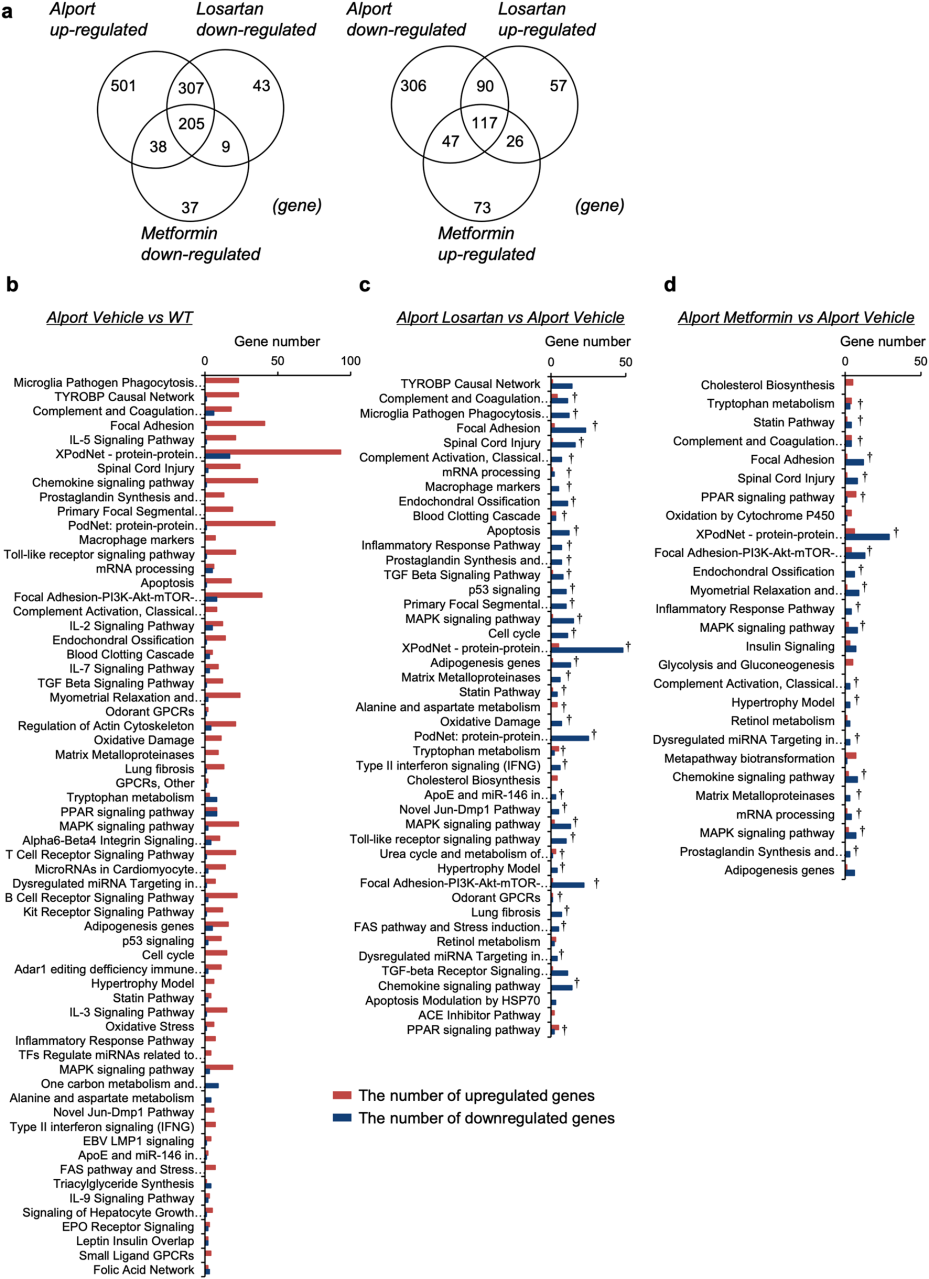
Transcriptome analysis reveals the comprehensive effects of metformin in kidney of *Col4a5* G5X Alport syndrome mice. (**a**) Venn diagram shows the number of fluctuated genes in three comparisons (WT vs Alport vehicle, Alport vehicle vs Alport metformin, Alport vehicle vs Alport losartan). (**b**–**d**) Pathway analysis of fluctuated genes in Alport vehicle (**b**), Alport losartan (**c**), and Alport metformin (**d**). Red and blue bars represent the number of upregulated and downregulated genes, respectively. Pathways are displayed in ascending order of *P* value (P < 0.05). †: Common pathways between Alport/vehicle vs Alport/losartan + metformin group.

To determine the molecular effects of metformin and losartan on Alport syndrome, we assessed the global mRNA expression profile in kidney tissues of WT and *Col4a5* G5X Alport syndrome mice treated with vehicle, metformin, or losartan for 10 weeks. Transcriptome analysis showed 1611 genes with differential expression between WT vs. Alport/vehicle, 854 genes between Alport/vehicle vs. Alport/losartan, and 552 genes between Alport/vehicle vs. Alport/metformin (fold change < − 2 or > 2, *P* value < 0.05, Fig. 4a). Supplementary Fig. S3 shows a heat map of these genes. We performed pathway analysis to characterize the altered genes. Genes with altered expression in the Alport/vehicle group were classified into 63 pathways, including complement associated, focal adhesion, inflammatory, and fibrosis pathways (Fig. 4b). In the Alport/losartan group, 45 pathways were altered with statistical significance, and 38 out of 45 significant pathways inversely correlated with the identified pathways in the Alport/vehicle group (Fig. 4c). In the Alport/metformin group, 27 pathways were altered with statistical significance, and 21 out of 27 significant pathways inversely correlated with identified pathways in the Alport/vehicle group (Fig. 4d). Metformin-specific altered genes were mainly classified into metabolic-related pathways such as PPAR signaling, insulin signaling, cholesterol biosynthesis, and glycolysis (Supplementary Fig. S4). In contrast, losartan-specific altered genes were mainly classified as inflammation-related pathways such as macrophage markers, complement, and MAPK signaling (Supplementary Fig. S5). These data also indicate that despite the similar phenotypic effects of losartan and metformin on CKD in *Col4a5* G5X Alport syndrome mice, these drugs influence different molecular signaling pathways.

### Metformin improved dysregulation of podocyte and metabolic pathways in glomeruli of *Col4a5* G5X Alport syndrome mice

**Figure 5:**
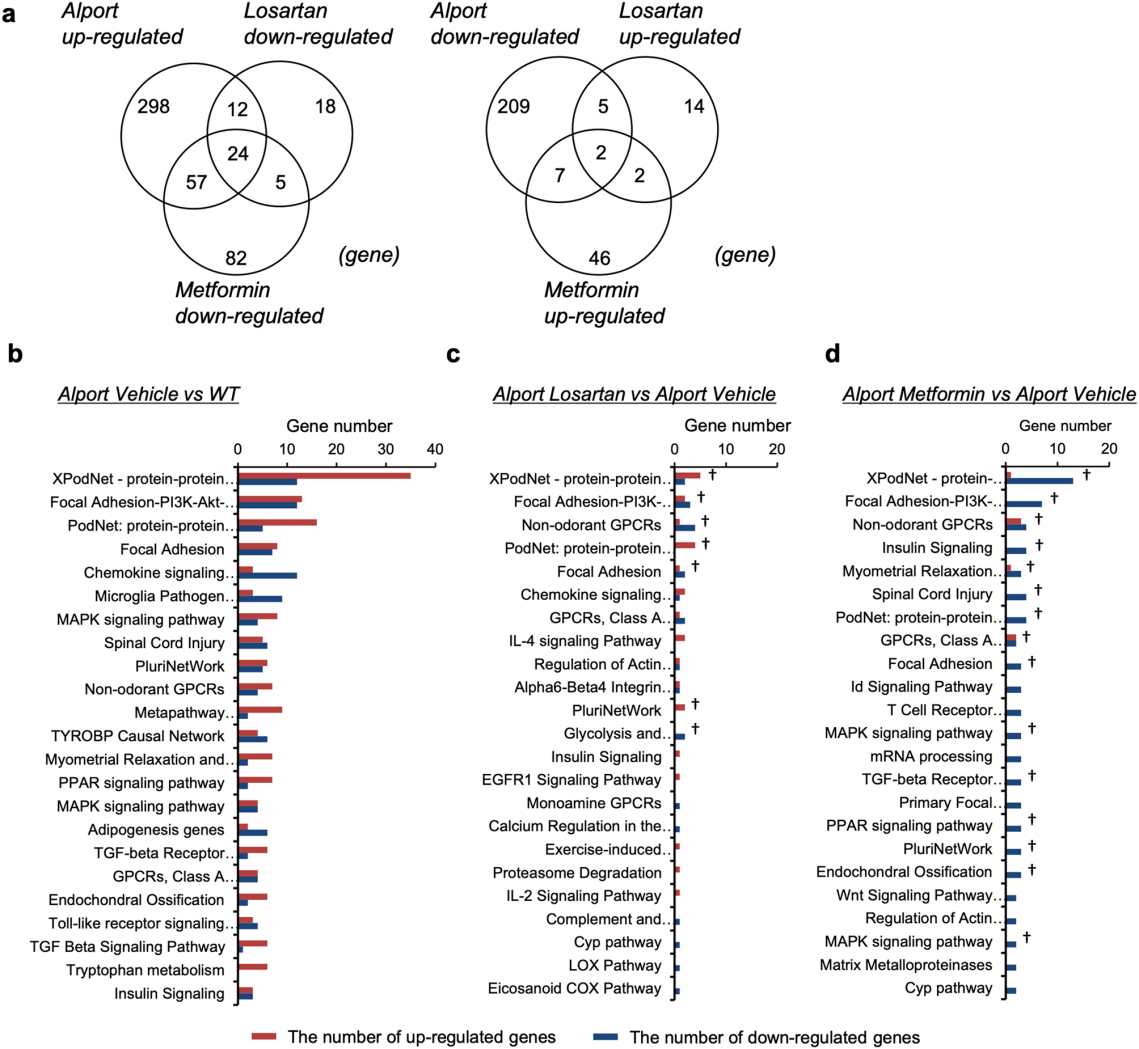
Transcriptome analysis reveals the comprehensive effects of metformin in glomeruli of *Col4a5* G5X Alport syndrome mice. (**a**) Venn diagram shows the number of fluctuated genes in three comparisons (WT vs. Alport vehicle, Alport vehicle vs. Alport metformin, Alport vehicle vs Alport losartan). (**b**–**d**) Pathway analysis of fluctuated genes in the different treatment groups, as indicated. Red and blue bars represent the number of upregulated and downregulated genes, respectively. Pathways are displayed in ascending order of *P* value (P < 0.05). †: Common pathways between Alport vehicle vs Alport losartan + metformin group.

To determine the molecular effect of metformin and losartan on glomeruli in *Col4a5* G5X Alport syndrome mice, we assessed the global mRNA expression profile in glomeruli of WT and Alport syndrome mice treated with vehicle, metformin, or losartan for 6 weeks. Transcriptome analysis showed 614 genes with differential expression between WT vs Alport/vehicle, 82 genes between Alport/vehicle vs. Alport/losartan, and 225 genes between Alport/vehicle vs Alport/metformin (fold change < − 2 or > 2, *P* value < 0.05, Fig. 5a). Supplementary Figs. S6–S8 show the heat map of these genes. We performed pathway analysis to characterize the altered genes. Unlike in the kidney tissue analysis (Fig. 4), microarray analysis of glomeruli identified podocyte-associated molecular pathways as the main abnormalities in the Alport/vehicle group (Fig. 5b). Moreover, dysregulation of metabolic pathways such as MAPK, PPAR, TGFβ and insulin signaling were identified. Losartan improved the dysregulation of podocyte-related pathways but did not improve the metabolic pathways (Fig. 5c). In contrast, metformin improved both abnormalities in podocyte-associated molecular pathways and metabolic pathways (Fig. 5d). These results suggest that losartan and metformin protected the glomerulus via different mechanisms.

### Metformin ameliorates the metabolic dysfunction in kidneys of *Col4a5* G5X Alport syndrome mice

Several reports demonstrated that metabolic processes are poorly regulated in CKD^26–28^, and our microarray analysis showed that the expression of metabolism-associated genes was dysregulated, including PI3K-Akt-mTOR, amino acid metabolism, and adipogenesis pathways (Fig. 4b). We performed metabolome analysis to investigate whether metformin affects intracellular metabolites in CKD. Results revealed differing levels of intercellular energetic metabolites, glycolysis-associated metabolites, and some amino acids and amino acid derivatives involved in metabolism, such as NADPH/NADP, cAMP, fructose 1-/6-phosphate, ribose 1-phosphate, proline, and *N*-acetylglutamic acid between WT and Alport syndrome/vehicle groups (Table 1). Notably, metformin or losartan normalized the NADPH/NADP ratio, glycolysis-, TCA cycle-, urea cycle-, and lipid metabolism-associated metabolites, and amino acids (Table 1). These results suggest that *Col4a5* G5X Alport syndrome mice had significantly altered metabolism, and that metformin or losartan modulated these metabolic changes in Alport syndrome.

**Table 1:** Altered metabolite in Alport vehicle (control), Alport losartan and Alport metformin.

Compound name	Pathway label	KEGG ID	Comparative analysis					
			Control vs WT		Losartan vs control		Metformin vs control	
			Ratio	*p*-value	Ratio	*p*-value	Ratio	*p*-value
Guanylate energy charge	No label	No ID	1.6	0.041*	0.7	0.028*	0.8	0.071^#^
NADPH/NADP+	No label	No ID	1.2	0.073^#^	0.8	0.121	0.9	0.049*
Total Pyr-related amino acids	No label	No ID	1.0	0.904	1.2	0.025*	1.0	0.909
Total acetyl CoA-related amino acids	No label	No ID	1.3	0.072^#^	1.0	0.779	1.0	0.404
Total succinyl CoA-related amino acids	No label	No ID	1.3	0.095^#^	1.1	0.448	1.0	0.774
SAM/SAH	No label	No ID	1.5	0.038*	0.8	0.127	0.9	0.468
Total amino acids	No label	No ID	1.0	0.390	1.1	0.025*	1.0	0.719
Total essential amino acids	No label	No ID	1.3	0.059^#^	1.1	0.535	0.9	0.458
Total non-essential amino acids	No label	No ID	1.0	0.701	1.1	0.019*	1.0	0.759
Total glucogenic amino acids	No label	No ID	1.0	0.498	1.1	0.021*	1.0	0.735
Total ketogenic amino acids	No label	No ID	1.3	0.068^#^	1.0	0.622	0.9	0.285
Total BCAA	No label	No ID	1.3	0.075^#^	1.1	0.590	1.0	0.939
Fischer's ratio	No label	No ID	1.1	0.024*	1.0	0.681	1.1	0.008**
Citrulline/ornithine	No label	No ID	1.6	0.053^#^	0.7	0.074^#^	0.8	0.184
NAD^+^	NAD+	C00003	0.6	0.007**	1.2	0.282	1.2	0.171
cAMP	cAMP	C00575	2.1	0.045*	0.6	0.094^#^	0.8	0.441
NADH	NADH	C00004	0.7	0.016*	1.1	0.324	1.1	0.467
UDP-glucose	UDP-Glc	C00029	1.3	0.056^#^	0.9	0.328	1.1	0.676
Uric acid	Uric acid	C00366	1.6	0.048*	0.8	0.147	0.9	0.532
NADP^+^	NADP+	C00006	0.7	0.027*	1.3	0.072^#^	1.2	0.098^#^
IMP	IMP	C00130	0.7	0.128	1.4	0.104	1.6	0.027*
Glucose 6-phosphate	G6P	C00092, C00668, C01172	1.4	0.110	0.7	0.056^#^	0.8	0.153
Fructose 6-phosphate	F6P	C00085, C05345	1.9	0.016*	0.6	0.030*	0.7	0.068^#^
Fructose 1-phosphate	D-F1P	C01094	2.3	0.095^#^	0.5	0.006**	0.8	0.305
Ribose 1-phosphate	R1P	C00620	0.6	0.039*	1.5	0.022*	1.4	0.100
Malonyl CoA	Malonyl-CoA	C00083	1.4	N.A.	0.7	0.002**	0.7	0.011*
Phosphocreatine	Phosphocreatine	C02305	0.2	0.001**	1.9	0.071^#^	1.9	0.416
Adenylosuccinic acid	Succinyl AMP	C03794	1.0	0.787	1.5	0.021*	1.5	0.008**
2,3-Diphosphoglyceric acid	Diphosphoglycerate	C01159	0.5	0.065^#^	1.4	0.285	1.1	0.709
Phosphoenolpyruvic acid	PEP	C00074	0.3	0.066^#^	2.1	0.060^#^	1.8	0.316
GTP	GTP	C00044	1.3	0.119	0.8	0.088^#^	0.9	0.075^#^
Glycerol 3-phosphate	Glycerol 3-phosphate	C00093	0.7	0.120	1.2	0.050*	1.3	0.232
*N*-Acetylglutamic acid	N-AcGlu	C00624	3.9	8.2E-04***	0.8	0.205	1.1	0.767
2-Hydroxyglutaric acid	2-Hydroxyglutaric acid	C01087, C02630, C03196	1.2	0.040*	0.9	0.201	1.1	0.223
Succinic acid	Succinic acid	C00042	0.8	0.041*	1.1	0.551	1.0	0.943
Citric acid	Citric acid	C00158	2.3	0.028*	0.6	0.044*	0.7	0.097^#^
*cis*-Aconitic acid	cis-Aconitic acid	C00417	2.5	0.038*	0.5	0.036*	0.7	0.376
Gly	Gly	C00037	1.0	0.626	1.2	0.033*	1.0	0.929
β-Ala	b-Ala	C00099	1.3	0.017*	1.0	0.422	1.0	0.819
*N*,*N*-Dimethylglycine	DMG	C01026	1.7	0.033*	0.8	0.222	0.7	0.076^#^
Ser	Ser	C00065, C00716, C00740	1.3	0.026*	1.1	0.262	0.9	0.269
Carnosine	Carnosine	C00386	1.9	0.013*	0.7	0.142	0.6	0.010**
Creatinine	Creatinine	C00791	2.5	0.031*	0.6	0.068^#^	0.8	0.429
Pro	Pro	C00148, C00763, C16435	2.8	0.004**	1.0	0.885	0.9	0.302
Val	Val	C00183, C06417, C16436	1.3	0.060^#^	1.1	0.594	1.0	0.958
Betaine	Betaine	C00719	1.8	6.0E-05***	1.0	0.836	1.1	0.039*
Thr	Thr	C00188, C00820	1.3	0.058^#^	1.1	0.284	0.9	0.249
Hydroxyproline	Hydroxyproline	C01015, C01157	1.4	0.008**	1.1	0.275	0.8	0.081^#^
Leu	Leu	C00123, C01570, C16439	1.3	0.062^#^	1.1	0.485	1.0	0.748
Asn	Asn	C00152, C01905, C16438	1.5	0.002**	1.0	0.519	0.8	0.043*
Gln	Gln	C00064, C00303, C00819	0.9	0.258	1.2	0.148	1.1	0.040*
Lys	Lys	C00047, C00739, C16440	1.3	0.051^#^	0.9	0.525	0.9	0.042*
Met	Met	C00073, C00855, C01733	1.0	0.986	1.4	0.027*	0.9	0.256
His	His	C00135, C00768, C06419	1.2	0.022*	1.1	0.634	1.1	0.477
Carnitine	Carnitine	C00318, C00487, C15025	0.9	0.271	1.3	0.037*	1.1	0.136
Phe	Phe	C00079, C02057, C02265	1.4	0.027*	1.0	0.769	0.9	0.243
Arg	Arg	C00062, C00792	1.2	0.067^#^	1.0	0.905	0.8	0.038*
Citrulline	Citrulline	C00327	2.2	0.025*	0.8	0.205	0.6	0.041*
*S*-Adenosylhomocysteine	SAH	C00021	0.6	0.020*	1.3	0.094^#^	1.1	0.584
Spermine	Spermine	C00750	0.5	0.022*	1.2	0.338	1.0	0.931
Trp	Trp	C00078, C00525, C00806	1.1	0.347	1.1	0.511	0.8	0.029*
Cystathionine	Cystathionine	C00542, C02291	0.8	0.234	1.5	0.054^#^	1.0	0.934
Argininosuccinic acid	ArgSuccinate	C03406	1.0	0.549	1.4	0.040*	0.9	0.553

*N.A.* Not available. Subject to calculation, but calculation was not possible due to insufficient data.

The ratio of the mean values between the two groups is calculated using the latter as the denominator.

The p-values was calculated by Welch's t-test (^#^< 0.1, *< 0.05, **< 0.01, ***< 0.001).

### Metformin protects against podocyte dysfunction in *Col4a5* G5X Alport syndrome mice

**Figure 6:**
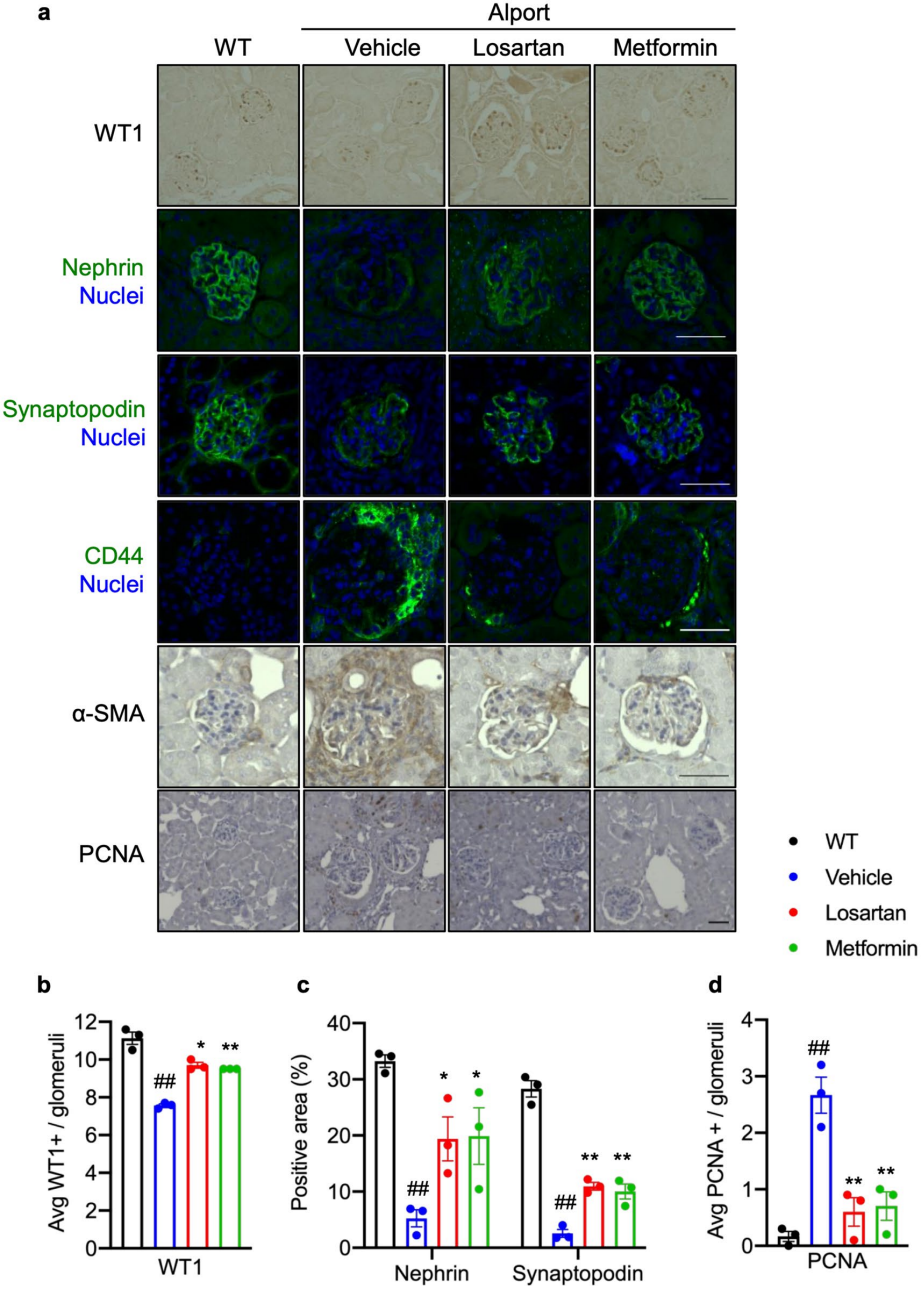
Metformin protects against podocyte dysfunction in *Col4a5* G5X Alport syndrome mice. (**a**) Visualization of indicated podocyte and glomerular cell proteins in renal tissue sections. Scale bars, 50 μm. (**b**) Quantification of WT1-positive cells in the glomerulus showed losartan or metformin protected against podocyte loss in late stage C57BL/6 *Col4a5* G5X vAlport syndrome mice. (**c**) Quantification of nephrin- and synaptopodin-positive area in the glomerulus showed that the decrease of these podocyte proteins in glomerulus was suppressed in losartan- or metformin-treated C57BL/6 *Col4a5* G5X Alport syndrome mice. (**d**) Quantification of PCNA-positive cells in the glomerulus showed the number of proliferating cells in glomerulus was decreased in losartan- or metformin-treated C57BL/6 *Col4a5* G5X Alport syndrome mice. Data are expressed as the means ± S.E. (n = 4 per group). *P* values were assessed by Dunnett’s test (^##^*P* < 0.01 vs WT. *P < 0.05, **P < 0.01 vs vehicle).

The podocyte is the most important cell in glomerular filtration. In glomerular disease and most types of CKD, podocyte functional proteins such as nephrin and synaptopodin are dysregulated leading to glomerular dysfunction^3,29^. A recent paper has shown that synaptopodin was dispensable in maintaining normal glomerular function but was protective in podocyte injury^30^. We found that metformin or losartan protected *Col4a5* G5X Alport syndrome mice against Alport syndrome-induced podocyte loss, which was evaluated by staining with the podocyte marker WT1 (Fig. 6a,b). Metformin or losartan also suppressed the down-regulation of nephrin and synaptopodin (Fig. 6a,c). In Alport syndrome and other glomerular diseases, glomerular cells, including parietal epithelial cells (PECs), are activated and proliferate^3,31^. Metformin or losartan suppressed CD44 expression, a marker of activated PEC^31^. Intraglomerular PCNA-positive (Fig. 6a,d) and periglomerular interstitial α-SMA-positive cells (Fig. 6a) were decreased in metformin or losartan-treated *Col4a5* G5X Alport syndrome mice. These data indicate that metformin or losartan prevents podocyte loss and dysregulation of PECs in *Col4a5* G5X Alport syndrome mice.

### Combining losartan and low-dose metformin extends the lifespan of Alport syndrome mice

**Figure 7:**
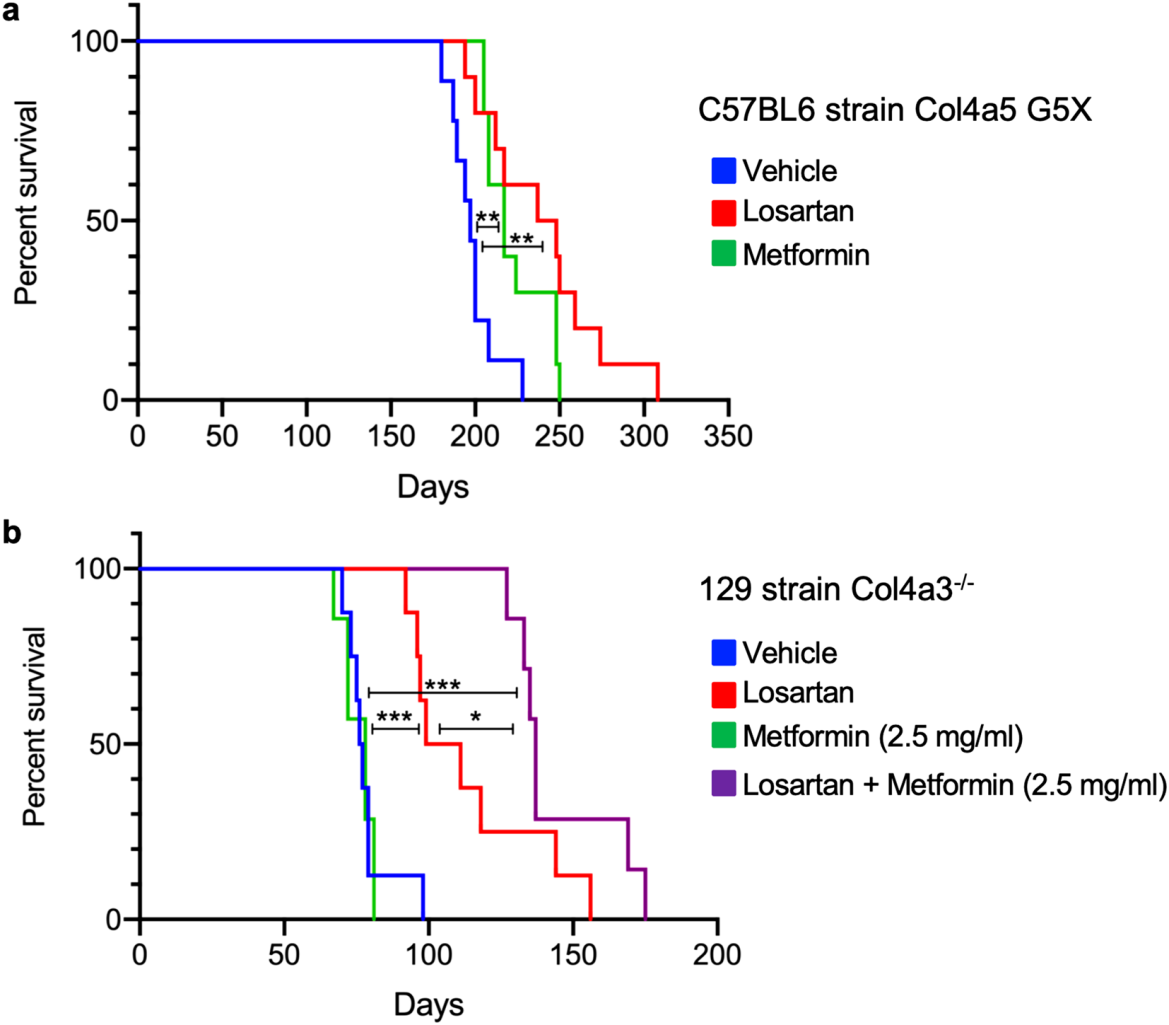
Combining losartan and low-dose metformin extends the lifespan of Alport syndrome mice. (**a**) The age at ESKD of C57BL/6 background *Col4a5* G5X Alport syndrome mice treated as indicated was determined. Losartan or metformin increased the age at ESKD. **P < 0.01 by Wilcoxon test (n = 9–10). (**b**) The age at ESKD of 129S1/SvImJ background *Col4a3*^−/−^ mice treated as indicated was determined. Low-dose metformin (2.5 mg/mL) was beneficial when combined with losartan. *P < 0.05, **P < 0.01, ***P < 0.005 by Wilcoxon test (n = 6–8).

Because metformin attenuated the proteinuria, inflammation and fibrosis in *Col4a5* G5X Alport syndrome mice, we investigated whether metformin ameliorates the onset of ESKD. We treated C57BL/6 background *Col4a5* G5X Alport syndrome mice with losartan or metformin via drinking water from 6 weeks old and monitored their survival, as presented in the schematic diagram of treatment (Fig. 1a and Supplementary Fig. S9a). Losartan or metformin extended the lifespan of *Col4a5* G5X Alport syndrome mice (Fig. 7a). Considering the different mechanism of action of metformin and losartan, we investigated whether their combination will increase mice survival. Contrarily, their combination caused acute toxicity and with this dosing, mice were dead in 2–3 weeks (data not shown). We do not know the cause of the toxicity, but we speculate that it is due to elevated blood levels of metformin although we could not measure the level of metformin in the blood. Because losartan decreases glomerular filtration rate (GFR), this may have increased the blood concentration of renally excreted metformin. To avoid the toxicity from high blood concentration, we used low-dose metformin for a further survival study. We treated 129S1/SvImJ background *Col4a3*^−/−^ Alport syndrome mice, which have a shorter lifespan than C57BL/6 background *Col4a5* G5X Alport syndrome mice, with losartan or metformin alone, or in combination, via drinking water from 4 weeks old (Supplementary Fig. S9b). Losartan (125 µg/mL) alone but not low-dose metformin (2.5 mg/mL) extended the lifespan. Notably, the combination of losartan (125 µg/mL) and low-dose metformin (2.5 mg/mL) increased the survival of *Col4a3*^−/−^ Alport syndrome mice more than losartan alone (Fig. 7b).

### Metformin protects against ADR-induced focal segmental glomerulosclerosis

We next investigated whether metformin has protective effects on another glomerular disease model by using ADR-induced focal segmental glomerulosclerosis (FSGS) mouse model. ADR nephropathy is also a well-characterized experimental glomerular disease model that exhibits proteinuria and glomerular injury^32,33^. Here, we found that metformin significantly reduced proteinuria and glomerular injury in ADR-induced FSGS mouse model (Supplementary Fig. S10a–c). Moreover, metformin induced p-AMPK and NRF2 protein expression and suppressed p-STAT3 expression (Supplementary Fig. S10d–g), indicating that metformin also has protective effect against ADR nephropathy.

## Discussion

This study demonstrated that metformin has protective effects against non-diabetic glomerular disease by suppressing proteinuria, renal inflammation, fibrosis, and glomerular injury in Alport syndrome mice. We also found that metformin ameliorated the proteinuria and glomerular injury in ADR-induced FSGS (Supplementary Fig. S10). To our knowledge, this is the first data showing that metformin alleviated some pathological phenotypes in Alport syndrome and ADR nephropathy. The effect of metformin on ADR-induced FSGS need further studies to delve deeper into the detailed molecular aspects, but these results provide proof of principle that metformin has potential in ameliorating another non-diabetic CKD not only Alport syndrome. These findings provide new insight concerning the pharmacological application of metformin for non-diabetic chronic glomerular diseases. Metformin is a drug approved to treat diabetes, but appears to target a number of signaling molecules. In our transcriptome analysis, metformin influenced not only metabolic regulators but also Alport syndrome-associated genes such as inflammation and matrix regulators. Moreover, metformin suppressed some exacerbating factors of kidney disease including p-STAT3^34,35^, p-mTOR^36,37^, and p-p38 MAPK^38,39^. Metformin increased the expression of p-AMPK^25,40^, p-p53^41,42^, and NRF2^43,44^, which are considered renal protective factors. The transcriptome analyses of kidney and of glomeruli were performed on 16-week-old and 12-week-old *Col4a5* G5X Alport syndrome mice, respectively. We chose these time points to dissect the direct effect of metformin on glomerulus and on the whole kidney. Glomerular injury is initiated and progresses at 8–12 weeks old, manifested by progressive proteinuria. At 16 weeks, renal inflammation and fibrosis occur in the kidney cortex^41^. These are the stages before significant glomerular sclerosis (12 weeks old) and cortex scarring (16 weeks old) would occur.

Glomerular diseases including Alport syndrome are primarily treated with renin–angiotensin system (RAS) inhibitors, immunosuppressive agents, and steroids^45,46^. However, most genetic glomerular diseases are resistant to immunosuppressive agents and steroid therapy^45,47,48^. In our study, the comparison between metformin and losartan, a commonly used angiotensin II type I receptor blocker, demonstrated similar protective effects of these drugs against Alport CKD. However, the comprehensive assessment at the molecular level revealed that these drugs targeted different pathways (Supplementary Figs. S4 and S5), and signaling molecules such as p-AMPK, p-p53, and NRF2 proteins were differentially regulated by metformin and losartan. Losartan, being an anti-hypertensive agent, exerted its protective effect by lowering intraglomerular pressure and reduced proteinuria without affecting the metabolic pathways. Metformin, an anti-diabetic agent that activates AMPK, mediated its glomerular protective effect via improvement of metabolic pathways in glomerular cells. These differences indicated that metformin ameliorated Alport syndrome via different mechanisms from those of losartan. In addition, we found that metformin changed gene expression more diversely than losartan in the transcriptome analysis of glomeruli (Fig. 5d). Considering the different pharmacological features and targets of losartan and metformin, combination therapy with metformin and RAS inhibitors could have additive effects on glomerular disease and CKD. Indeed, the combination of low-dose metformin and losartan extended the lifespan of Alport syndrome mice compared with losartan alone.

Until recently, metformin was not recommended for patients with kidney disease due to the risk of lactic acidosis^24^. However, accumulated lines of evidence have shown that the incidence of lactic acidosis was lower in patients treated with metformin than previously thought^49,50^. The Food and Drug Administration has now approved metformin for use in certain patients with reduced kidney function^17,51^. Here, due to technical limitation, we were not able to measure the level of metformin in the blood, but we assessed the potential side effects of metformin and found that it did not induce low blood glucose and lactic acidosis (Supplementary Fig. S2). Our data and previous reports indicate that metformin can be considered a novel treatment option for chronic glomerular disease and CKD. However, because metformin is known to be excreted via urine^52^, GFR needs to be closely monitored. Metformin is a drug prescribed for children, and it is one of the more affordable drugs. Our finding that metformin could ameliorate Alport CKD pathologies presents a promising avenue for pharmacological treatment of Alport syndrome, pediatric glomerular disease, and CKD, and supports the current trend toward reconsidering the use of metformin for patients with kidney disease.

## Materials and methods

### Animals

The C57BL/6 background X-linked Alport syndrome mouse model (*Col4a5* < tm1Yseg > G5X mutant) was developed as previously described^20^. *Col4a5* gene is located on the X chromosome and the G5X mutation is X-linked inheritance. The C57BL/6 *Col4a5* G5X Alport syndrome mice mouse model is well characterized, showing progressive glomerular disease and phenotypes of CKD, including persistent proteinuria, renal inflammation, and fibrosis^20^. These mice were obtained from The Jackson Laboratory (Bar Harbor, ME, USA). For experiments using C57BL/6 *Col4a5* G5X Alport syndrome mice, we applied artificial reproductive techniques established by the Center for Animal Resources and Development (CARD, Kumamoto University) to obtain a sufficient number of mice^53^. Age-matched 6-week-old C57BL6J wild type (WT) mice were used as controls to compare the phenotypes of C57BL/6 *Col4a5* G5X Alport syndrome mice. Metformin was orally administered via drinking water at a dose of 5 mg/mL to C57BL/6 *Col4a5* G5X Alport syndrome mice starting at 6 weeks old to 11 weeks old. Twelve-week-old mice were either sacrificed to harvest glomeruli for transcriptome analysis of glomeruli, or were continually treated with metformin at a dose of 2.5 mg/mL. At 16 weeks old, kidney cortex was harvested for transcriptome analysis. For histological analysis, mice were treated with metformin similarly as above until 20 weeks old when renal organ was harvested for further analysis. Losartan was administered via drinking water at a dose of 250 µg/mL to 6- to 11-week-old C57BL/6 *Col4a5* G5X Alport syndrome mice, and 125 µg/mL to 12- to 20-week-old C57BL/6 *Col4a5* G5X Alport syndrome mice. The schematic diagram of the treatments is presented in Fig. 1a and Supplementary Fig. S9a. The dosage of metformin used was based on a previous study^54,55^. The water containing these compounds was replenished twice per week. In all experiments, male mice were used to avoid sex differences. Metformin and losartan were obtained from Wako Pure Chemical Industries (Tokyo, Japan).

The 129S1/SvImJ background autosomal recessive Alport syndrome mice (*Col4a3*^−/−^) was developed as previously described^56^. For survival studies, low-dose metformin (2.5 mg/mL), losartan (125 µg/mL) or the combination of these were orally administered via drinking water to 4-week-old 129S1/SvImJ *Col4a3*^−/−^ Alport syndrome mice. A schematic diagram of the treatments is shown in Supplementary Fig. S9b. The Animal Welfare Committee of Kumamoto University (#A28-059) approved all animal experiments using C57BL/6 *Col4a5* G5X Alport syndrome mice. All animal experiments using 129S1/SvImJ *Col4a3*^−/−^ Alport syndrome conformed with the National Institutes of Health Guide for the Care and Use of Laboratory Animals and were approved by the Washington University Institutional Animal Studies Committee. The study was carried out in compliance with the ARRIVE guidelines^57^.

### Kidney function analysis

Urine samples were collected for 24 h using metabolic cages (As One, Osaka, Japan). Urinary protein and albumin were measured by the Bradford method (Bio-Rad Laboratories, CA, USA) and Albuwell M (Exocell Inc, PA, USA), respectively. Urinary creatinine was measured by Jaffe’s method (Wako Pure Chemical Industries, Tokyo, Japan). Urinary protein and albumin concentrations were normalized with urinary creatinine concentration. Mouse blood samples were collected as previously described^35^. Serum creatinine was measured using Fuji Dri Chem CRE-PΙΙΙ (Fujifilm, Tokyo, Japan).

### Histological analysis and immunostaining

Periodic acid-Schiff (PAS) staining and Masson’s trichrome (MT) staining of paraffin kidney sections were performed by standard methods. Glomerular injury scores were quantified as previously described^35^. For tubulointerstitial fibrosis, MT-positive area was quantified using BX-X700 microscope and image analysis software (KEYENCE, Osaka, Japan). MT-stained area vs unstained area was calculated, and presented as % fibrotic region. For immunofluorescence, kidney tissues were frozen with liquid nitrogen in optimal cutting temperature (OCT) compound. Frozen tissues were sliced at 6-µm thickness, and processed as reported previously^41^. For immunohistochemistry, paraffin-embedded tissues were sliced at 4-µm thickness and handled as previously described^35^. For immunochemistry, paraffin-embedded tissues were sliced at 4-µm thickness. Samples were reacted with the indicated antibodies. Nephrin- and Synaptopodin-positive areas in glomerulus were quantified using BX-X700 microscope and image analysis software (KEYENCE, Osaka, Japan). Positive-stained area vs glomerular area was calculated, and presented as % positive area.

### Glomerular injury scoring

To determine glomerular injury score, as described previously^35^, more than 100 PAS-stained random glomeruli per mouse (n = 4 mice) were examined, and scored from 0 to 4 (0, no lesion; 1, expansion of mesangial area; 2, expansion of Bowman’s epithelial cells and adhesion of glomeruli and Bowman’s capsule; 3, sclerotic area in 50–75% of glomerulus; 4, sclerotic area in 75–100% of glomerulus). Double blind scoring was performed and values were computed and presented in a graph as percentage.

### Western blotting analysis

Kidney tissues were washed with PBS and lysed in radioimmunoprecipitation (RIPA) buffer (50 mM Tris–HCl, 150 mM NaCl, 1 mg/mL sodium deoxycholate, and 1% NP-40, containing 1% protease inhibitor cocktail (Sigma-Aldrich), 2 mM sodium vanadate, and 100 mM sodium fluoride). Lysates were analyzed by immunoblotting using standard methods as described previously^41^. Primary and secondary antibodies were diluted in CanGet Signal immunoreaction Enhancer Solutions 1 and 2, respectively (Toyobo, Osaka, Japan). SuperSignal WestPico chemiluminescence substrate (Thermo Fisher Scientific) and Amersham ECL prime (GE Healthcare) were used for visualizing the blots.

### Antibodies

For immunofluorescence staining of kidney tissues, the antibodies used were guinea pig anti-Nephrin (clone GP-N2, Progen, Heidelberg, Germany), mouse anti-synaptopodin (clone G1D4, Progen), and rat anti-mouse CD44 (clone IM7, BD Biosciences). For immunohistochemistry, the antibodies used were rat anti-F4/80 (ab6640, Abcam, Cambridge, UK), rabbit anti-WT1 (clone C-19, Santa Cruz Biotechnology, CA, USA), rabbit anti-α-SMA (ab5694, Abcam), and mouse anti-PCNA (clone PC10, DAKO). All antibodies were diluted at 1:100 in DAKO antibody diluent (Agilent, CA, USA). The following polyclonal rabbit antibodies used for primary reaction of immunoblots were obtained from Cell Signaling Technology (MA, USA): anti-phosphorylated Smad1/5/8 (#95115), anti-phosphorylated AMPK (#2531), anti-AMPK (#2532), anti-phosphorylated p38 (#4511), anti-p38 (#9212), anti-phosphorylated ERK (#4370), anti-ERK (#4695), anti-phosphorylated STAT3 (#9145), anti-phosphorylated p53 (#9284), and anti-phosphorylated mTOR (#2971). Rabbit antibodies anti-α-SMA (ab5694) and anti-NRF2 (ab31163) were from Abcam. Rabbit antibodies anti-STAT3 (clone C-20), anti-p53 (clone FL393), and anti-vinculin (clone H-300) were obtained from Santa Cruz Biotechnology.

### Quantitative RT-PCR

RNA samples were isolated from kidney tissues, which were soaked in RNAlater (Thermo Fisher Scientific, MA, USA) for 24 h using RNAiso plus (Takara Bio, Shiga, Japan). Quantitative RT-PCR was performed as previously described^35^. The primer sequences are shown in Table 2.

**Table 2:** Primers used in qRT-PCR.

Gene	Sense	Antisense
**Mouse**		
*IL-6*	5′-GAGGATACCACTCCCAACAGACC-3′	5′-AAGTGCATCATCGTTGTTCATACA-3′
*IL-1β*	5′-GCTGAAAGCTCTCCACCTCAATG-3′	5′-TGTCGTTGCTTGGTTCT CCTTG-3′
*IL-8 (KC)*	5′-TGTCAGTGCCTGCAGACCAT-3′	5′-GAGCCTTAGTTTGGACAGGATCTG-3′
*Mcp1*	5′-GAAGCTGTAGTTTTTGTCACCAAG-3′	5′-AGGTAGTGGATGCATTAGCTTCA-3′
*Mmp12*	5′-CATGAAGCGTGAGGATGTAGAC-3′	5′-TGGGCTAGTGTACCACCTTTG-3′
*Mmp9*	5′-GGACCCGAAGCGGACATTG-3′	5′-CGTCGTCGAAATGGGCATCT-3′
*α-Sma*	5′-CCCAGACATCAGGGAGTAATGG-3′	5′-TCTATCGGATACTTCAGCGTCA-3′
*Tgf-β*	5′-CACCTGCAAGACCATCGACAT-3′	5′-GAGCCTTAGTTTGGACAGGATCTG-3′
*Lysozyme*	5′-CCAGTGTCACGAGGCATTCA-3′	5′-TGATAACAGGCTCATCTGTCTCA-3′
*Gapdh*	5′-CCTGGAGAAACCTGCCAAGTATG-3′	5′-GGTCCTCAGTGTAGCCCAAGATG-3′

### Transcriptome analysis

The isolation and purification of RNA samples from kidney tissues and glomeruli were performed using RNeasy Mini Kit (QIAGEN) according to the manufacturer’s recommended protocol. DNA was removed from the samples using RNase-free DNase Set (QIAGEN). The purity and integrity of isolated RNA was checked by Epoch Microplate Spectrophotometer (BioTek) and agarose gel electrophoresis. One-hundred ng of total RNA was used to generate amplified and biotinylated sense strand cDNA according to the GeneChip WT PLUS Reagent Kit User Manual (Thermo Fisher Scientific). cDNA was hybridized to the Clariom S Array, Mouse (Thermo Fisher Scientific) and scanned with GeneChip Scanner 3000 7G (Affymetrix).

Gene expression analysis was performed using Robust Multi-array Average (RMA) algorithm of Transcriptome Analysis Console 4.0 software (Thermo Fisher Scientific), as previously described^58^. Differentially expressed genes, fold change (log2) of ≧ 2 or ≦ − 2, p < 0.05 (WT vs Alport vehicle, Alport vehicle vs Alport metformin, Alport vehicle vs Alport losartan) were picked up and subjected to Venn diagram, heatmap and pathway analysis (WikiPathways database). The microarray data set was deposited in Gene Expression Omnibus with a registration number of GSE109861.

### Metabolome analysis

Metabolome analysis was performed using C-Scope [Human Metabolome Technologies (HMT), Yamagata, Japan] according to the recommended protocol. In brief, kidney tissue samples were collected from WT and metformin- or losartan-treated C57BL/6 *Col4a5* G5X Alport syndrome mice, and quickly frozen in liquid nitrogen. Frozen kidney tissues were treated with 50% (v/v) acetonitrile water solution. Then, 10 µM HMT internal standard solution was added and kidney tissues were freeze crushed. After tissue crushing, samples were centrifuge at 2300*g*, 4 °C for 5 min. Supernatants were collected and filtered using Ultrafree MC PLHCC 5 kDa (HMT, Yamagata, Japan). Filtered sample solutions were dried and resuspended with deionized water. Metabolome analysis was performed by capillary electrophoresis time-of-flight mass spectrometry (CE-TOFMS) and capillary electrophoresis tandem mass spectrometry (CE-QqQMS; CE-MS/MS).

### Statistical analysis

All data are presented as mean ± S.E. The statistical significance of the difference between two groups was assessed using Student’s *t* test. For more than three groups, statistical difference among groups was assessed using one-way analysis of variance (ANOVA) with Dunnett’s test. Statistical analysis was performed using JMP13 Statistical Discovery (SAS Institute Inc., Cary, NC, USA).

## Supplementary Information

Supplementary Information.

## Data Availability

All data generated or analyzed during this study are included in this published article and its Supplementary Information files.
