# Supplementary material for: Metformin ameliorates the severity of experimental Alport syndrome
Source: Sci Rep. 2021 Mar 29;11:7053. doi: 10.1038/s41598-021-86109-1 (PMC8007696; doi:10.1038/s41598-021-86109-1)
Supplement: Supplementary file 1 — Supplementary Information. [file 41598_2021_86109_MOESM1_ESM.pdf]

Supplementary Information

Metformin Ameliorates the Severity of Experimental Alport Syndrome

Kohei Omachi[1,2,7,#], Shota Kaseda[1,2,#], Tsubasa Yokota[1], Misato Kamura[1,2], Keisuke Teramoto[1,2], Jun Kuwazuru[1], Haruka Kojima[1], Hirofumi Nohara[1,2], Kosuke Koyama[1], Sumio Ohtsuki[3], Shogo Misumi[4], Toru Takeo[5], Naomi Nakagata[5], Jian-Dong Li[6], Tsuyoshi Shuto[1], Mary Ann Suico[1], Jeffrey H. Miner[7] and Hirofumi Kai[1,2,*]

[1]Department of Molecular Medicine; [2]Program for Leading Graduate School "HIGO (Health Life Science: Interdisciplinary and Glocal Oriented) Program"; [3]Department of Pharmaceutical Microbiology; [4]Department of Environmental and Molecular Health Sciences, Graduate School of Pharmaceutical Sciences, Kumamoto University, 5-1 Oe-honmachi, Chuo-ku, Kumamoto 862-0973, Japan; [5]Division of Reproductive Engineering, Center for Animal Resources and Development (CARD), Kumamoto University, 2-2-1 Honjo, Chuo-ku, Kumamoto 860-0811, Japan; [6]Center for Inflammation, Immunity & Infection, Institute for Biomedical Sciences, Georgia State University, Petit Science Center, 100 Piedmont Ave SE, Atlanta GA30303, USA, [7]Division of Nephrology, Washington University School of Medicine, 4523 Clayton Ave., St. Louis, MO, 63110, United States.

Supplementary Methods

For the ADR-induced nephritis model, BALB/c mice were given a single injection of Adriamycin (ADR,10 mg/kg) via retro-orbital injection. BALB/c mice were obtained from CLEA, Inc. (Tokyo, Japan). Metformin (5 mg/ml in drinking water) or vehicle was orally given to the ADR nephritis mice from day 1 after ADR injection until day 28. Metformin was purchased from Wako Pure Chemical Industries (Tokyo, Japan) and ADR was purchased from Sigma-Aldrich (St Louis, MO, USA).

Supplementary Figure S1

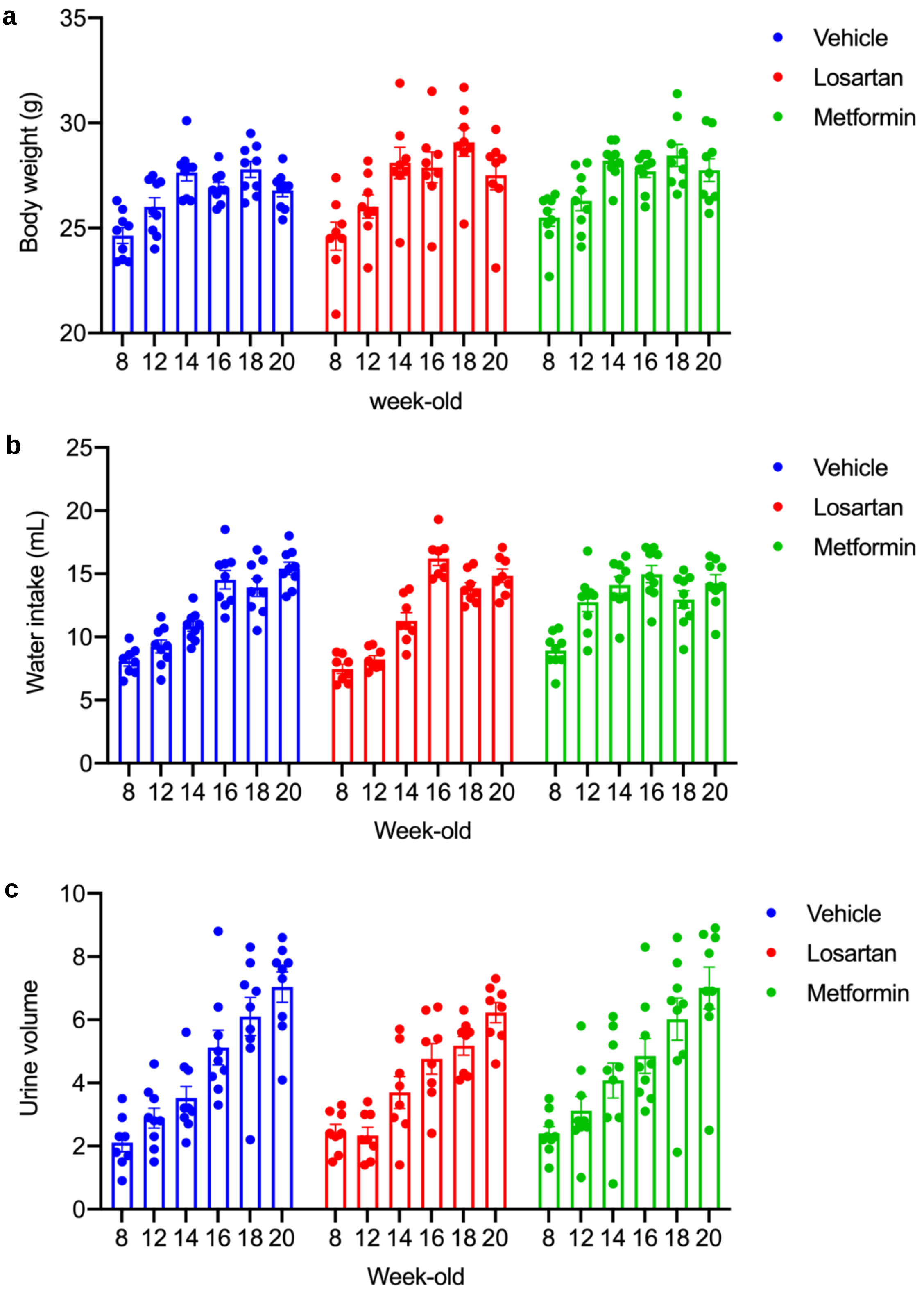

Supplementary Figure S1. Metformin did not affect the body weight, water intake and urine volume

(**a**) Body weight, (**b**) water intake and (**c**) urine volume were measured every four weeks. Urine volume was measured using metabolic cages for 24 hr. Bars indicate the mean ± S.E. (n=8-9).

Supplementary Figure S2

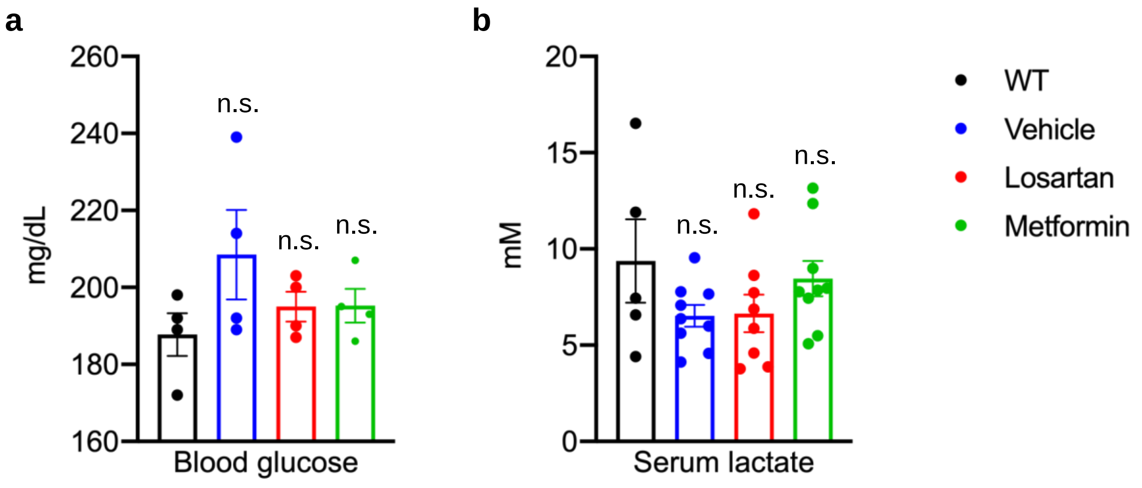

Supplementary Figure S2. Metformin did not affect the blood glucose and serum lactate levels

(**a**) Blood glucose level was measured at 8 weeks of treatment with ACCU-CHEK Compact (Roche Diagnostic, Mannheim, Germany). (**b**) Serum lactate level was measured at 10 weeks of treatment using L-Lactate Assay kit (Ab65331, Abcam). Bars indicate the mean ± S.E. (n = 4 per group).

Supplementary Figure S3

a

Inflammation

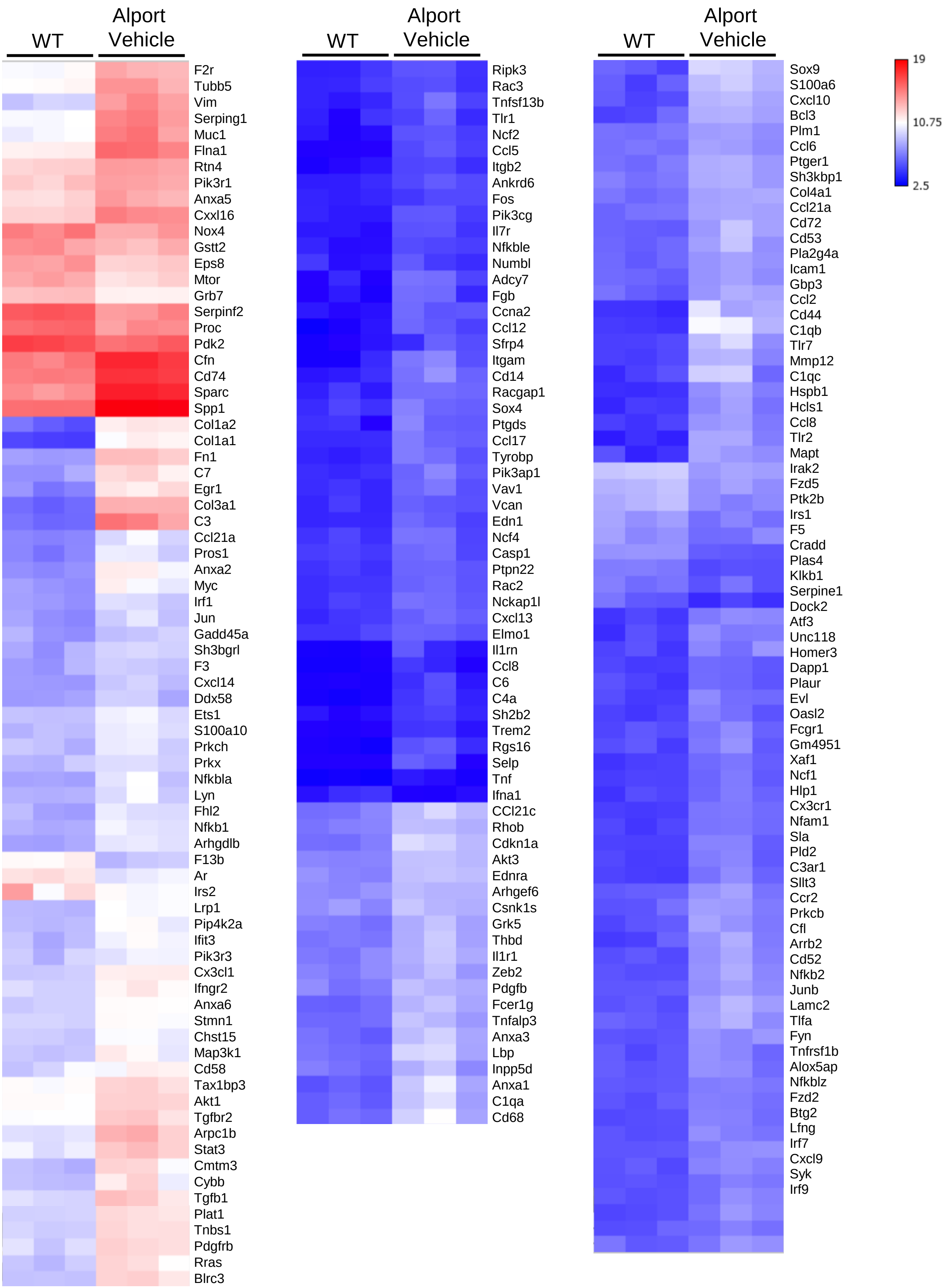

(Continue to the next page)

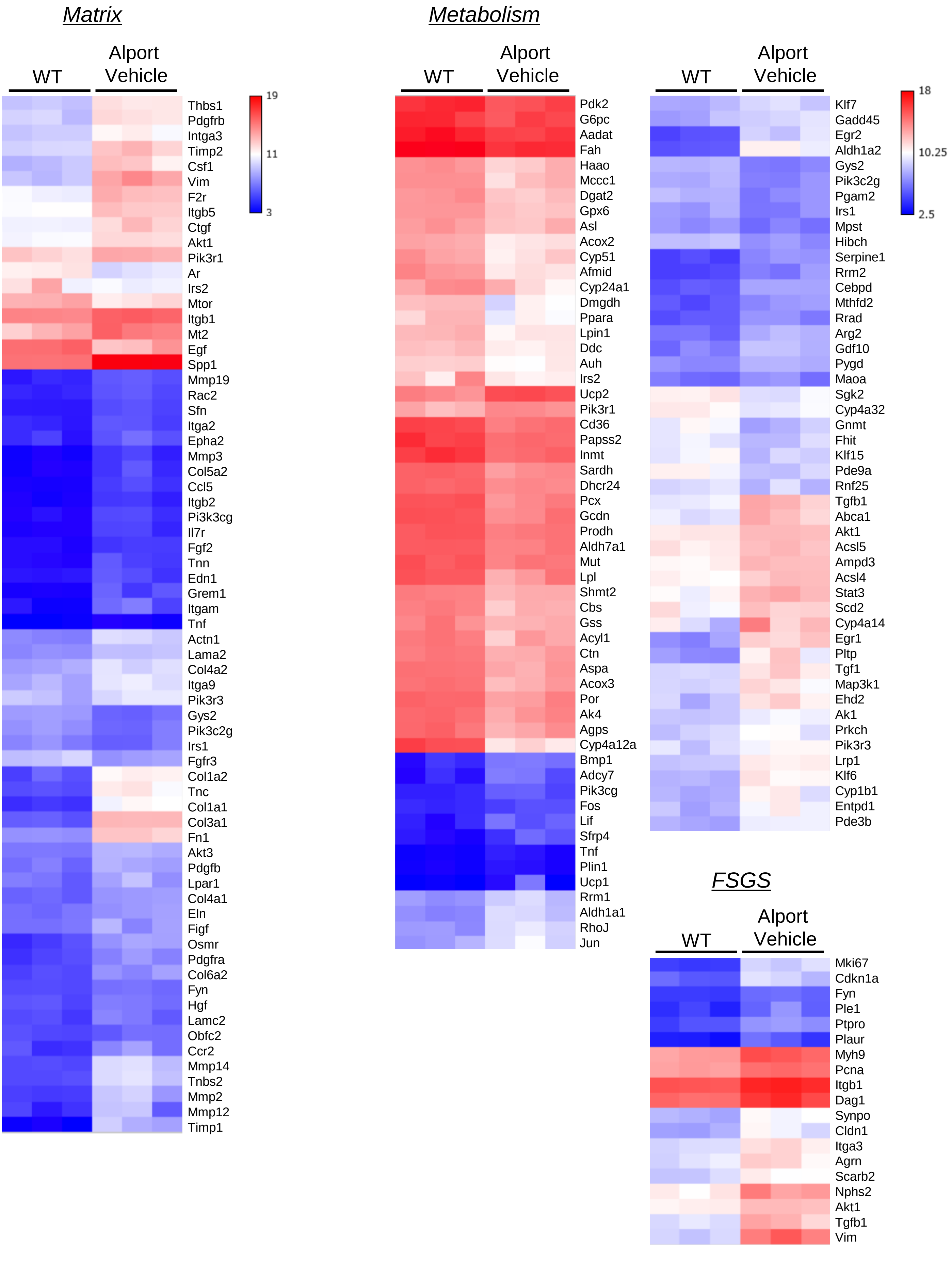

(Continue to the next page)

b

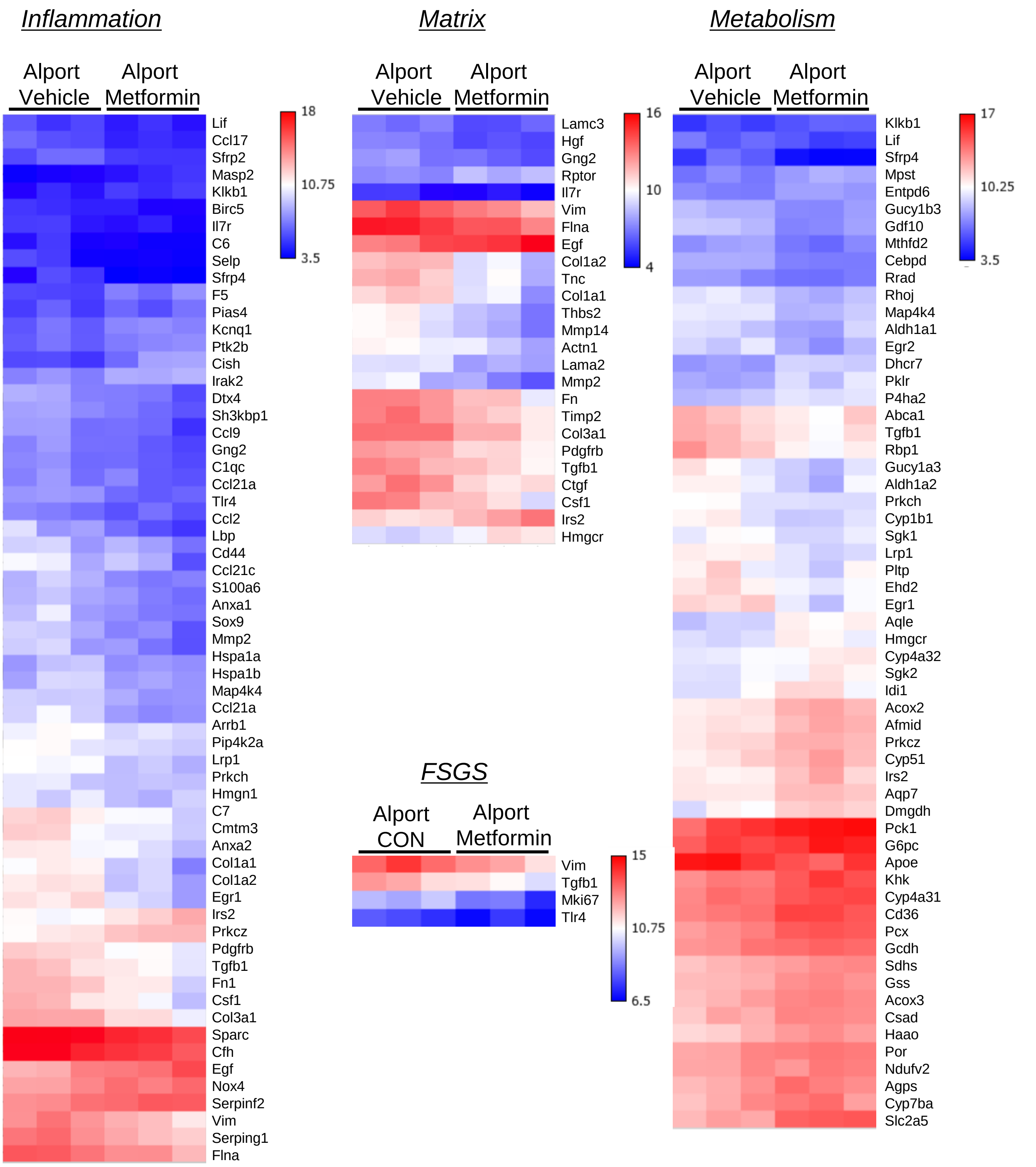

(Continue to the next page)

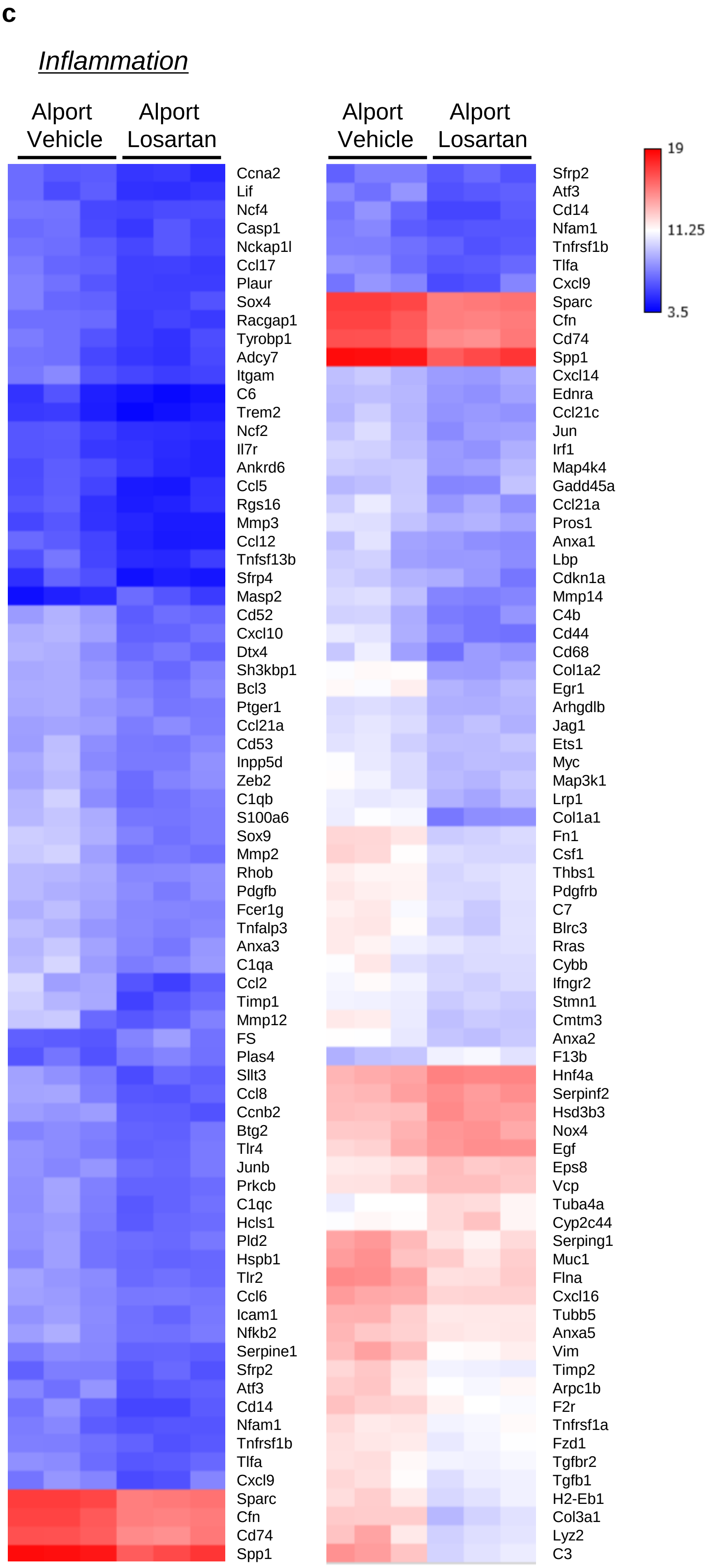

(Continue to the next page)

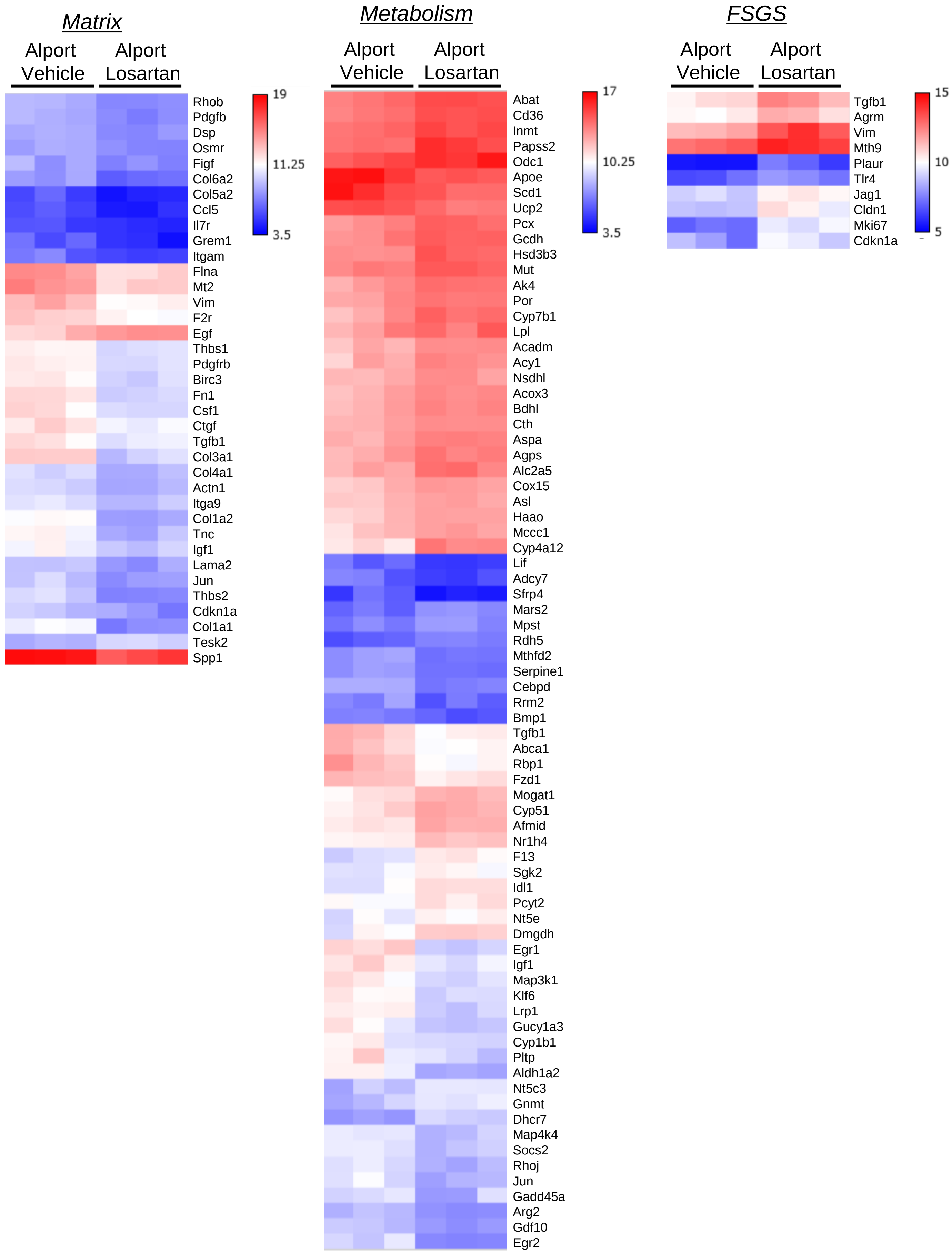

Supplementary Figure S3. Transcriptome analysis of kidney revealed the comprehensive effects of metformin on CKD in *Col4a5* G5X-Alport syndrome mice

Heat map shows the fluctuated genes associated with inflammation, matrix, metabolism and FSGS in three comparisons **(a:** WT vs Alport vehicle, **b**: Alport vehicle vs Alport metformin, **c**: Alport vehicle vs Alport losartan)

Supplementary Figure S4

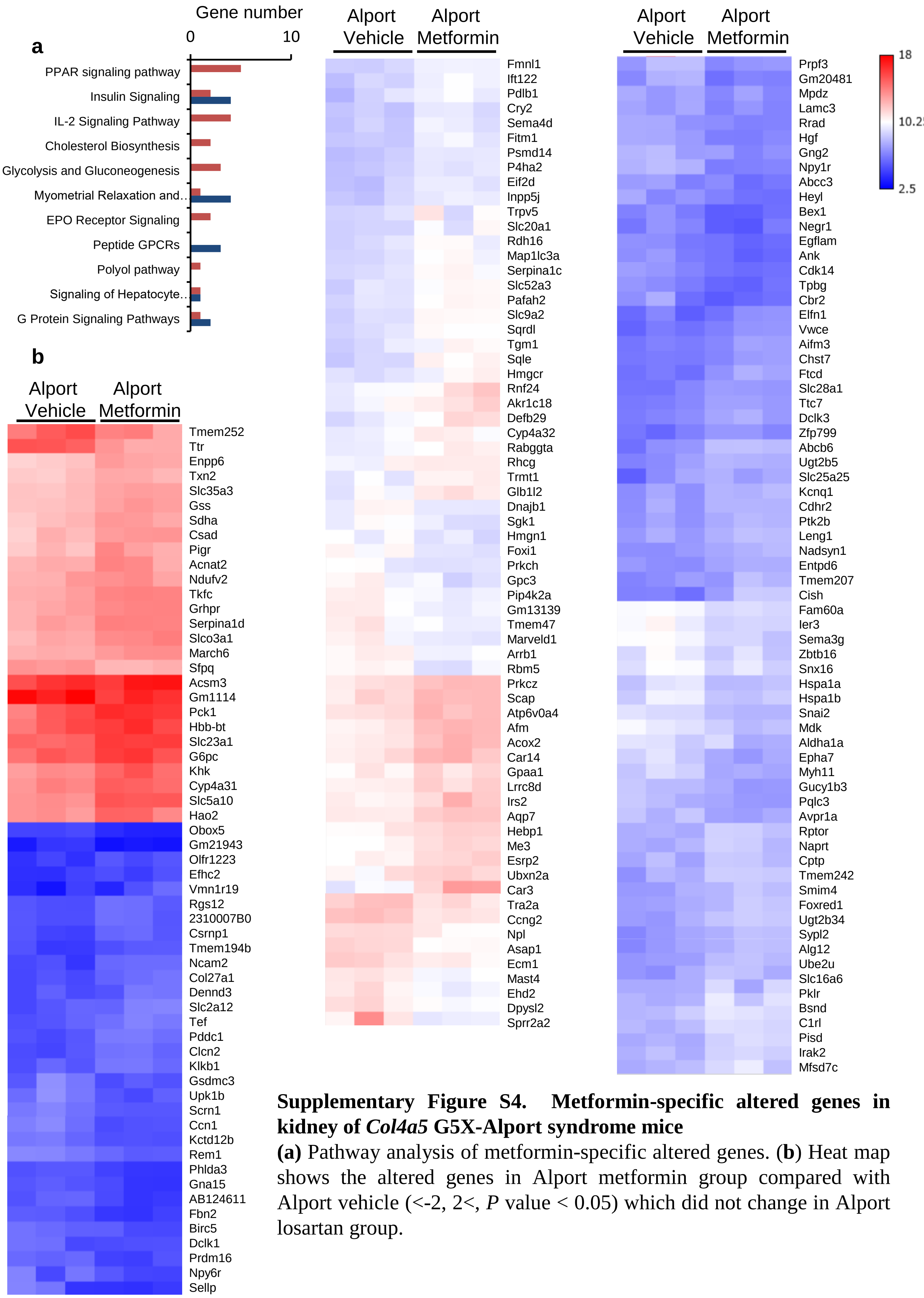

Supplementary Figure S4. Metformin-specific altered genes in kidney of *Col4a5* G5X-Alport syndrome mice

(a) Pathway analysis of metformin-specific altered genes. **(b)** Heat map shows the altered genes in Alport metformin group compared with Alport vehicle (<-2, 2<, P value < 0.05) which did not change in Alport losartan group.

Supplementary Figure S5

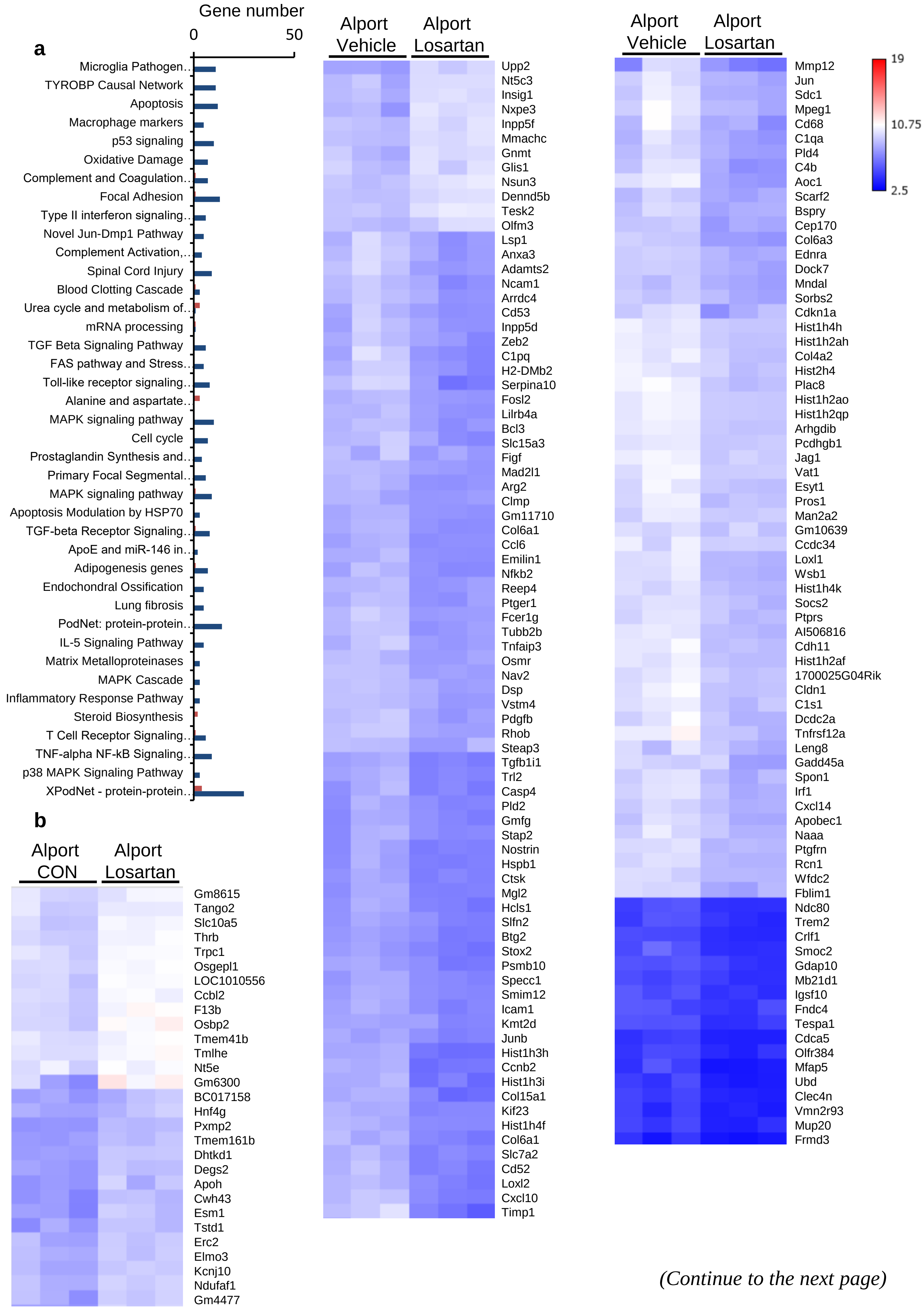

(Continue to the next page)

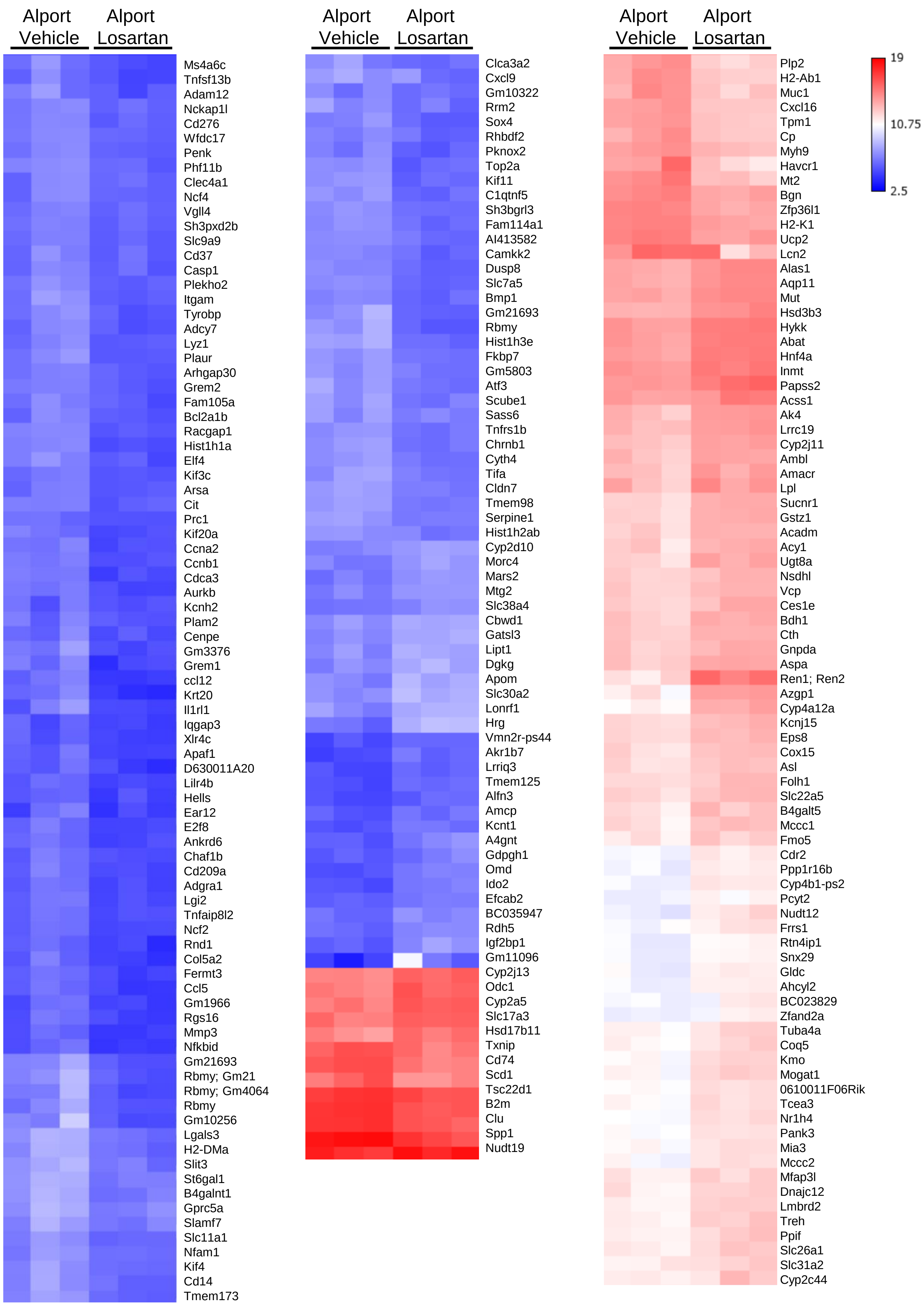

(Continue to the next page)

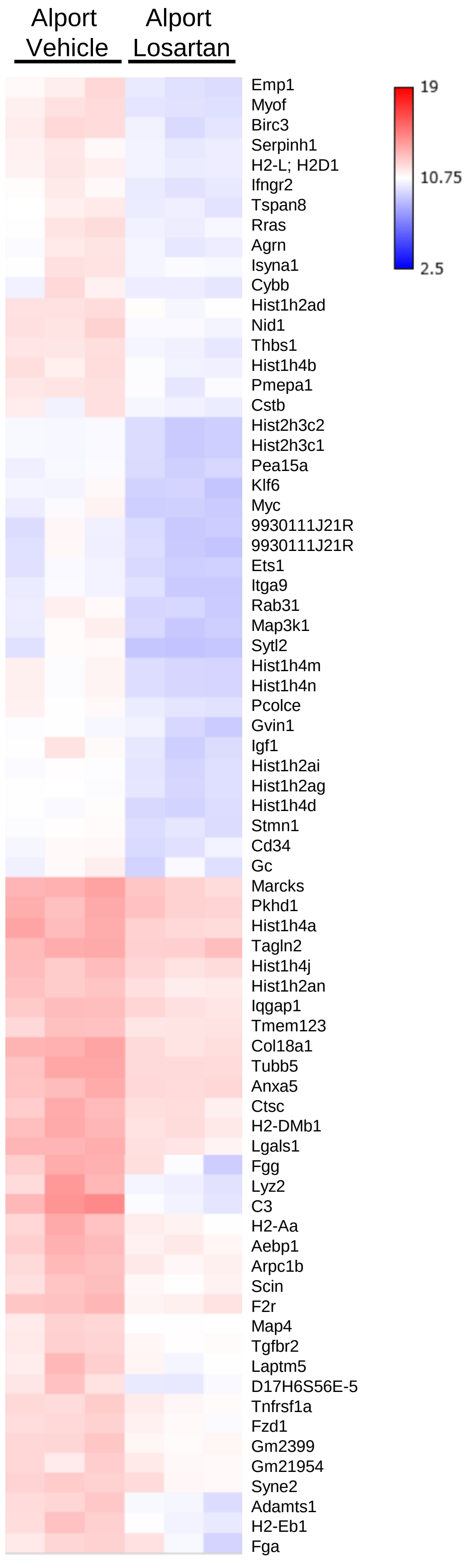

Supplementary Figure S5. Losartan-specific altered genes in kidney of *Col4a5* G5X-Alport syndrome mice

(a) Pathway analysis of metformin-specific altered genes. **(b)** Heat map shows the altered genes in Alport losartan group compared with Alport vehicle (<-2, 2<, P value < 0.05) which did not change in Alport metformin group.

Supplementary Figure S6

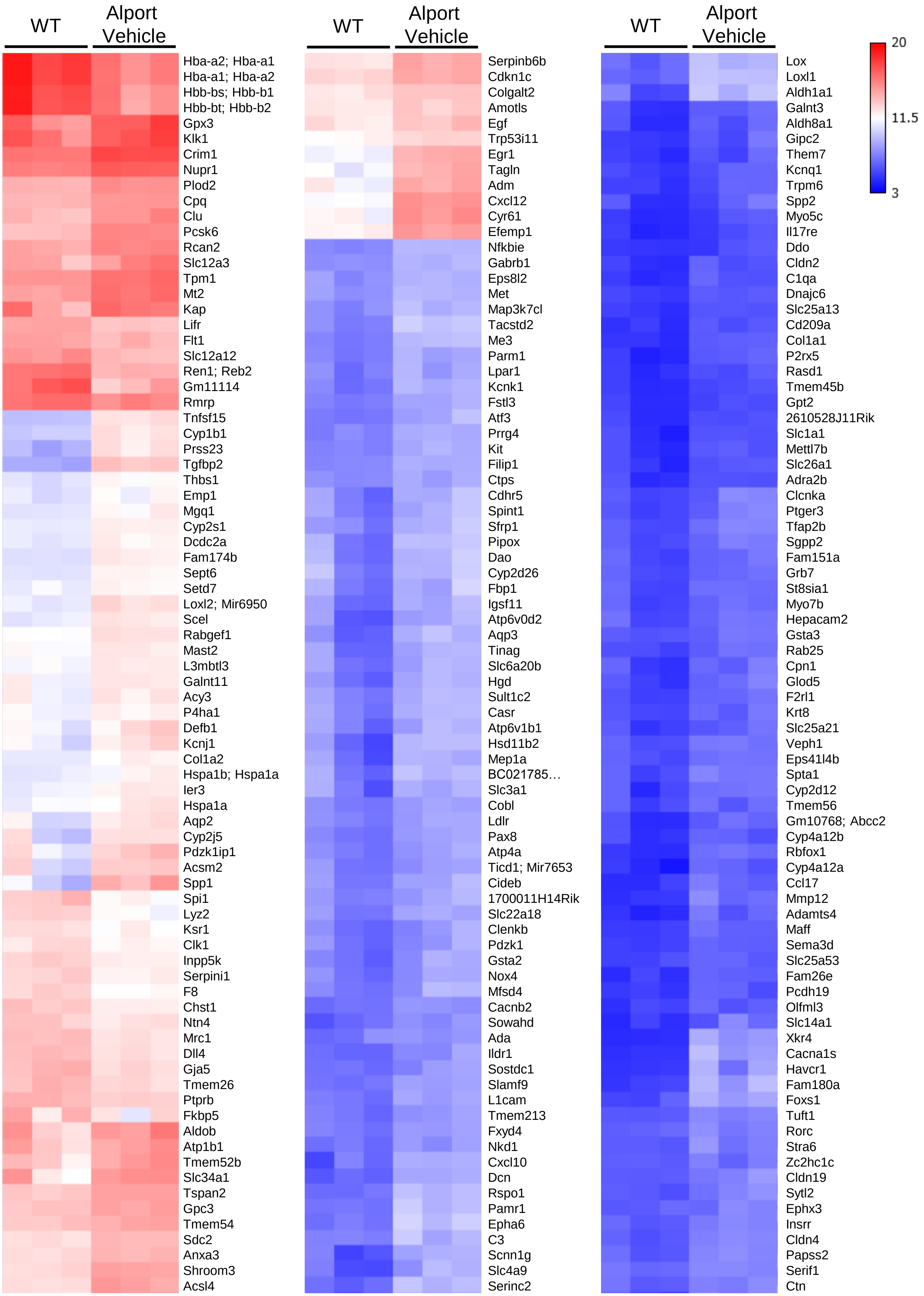

(Continue to the next page)

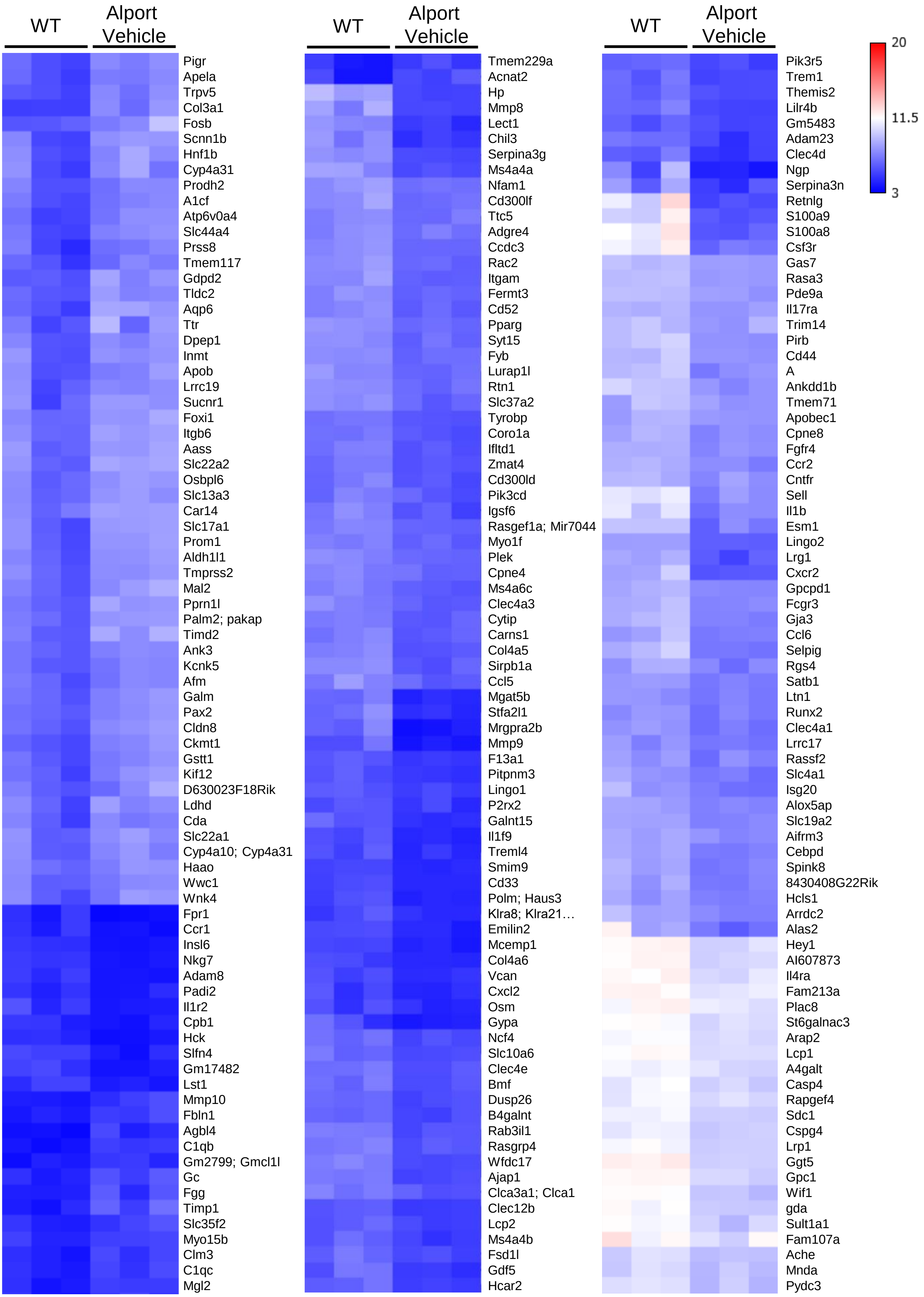

(Continue to the next page)

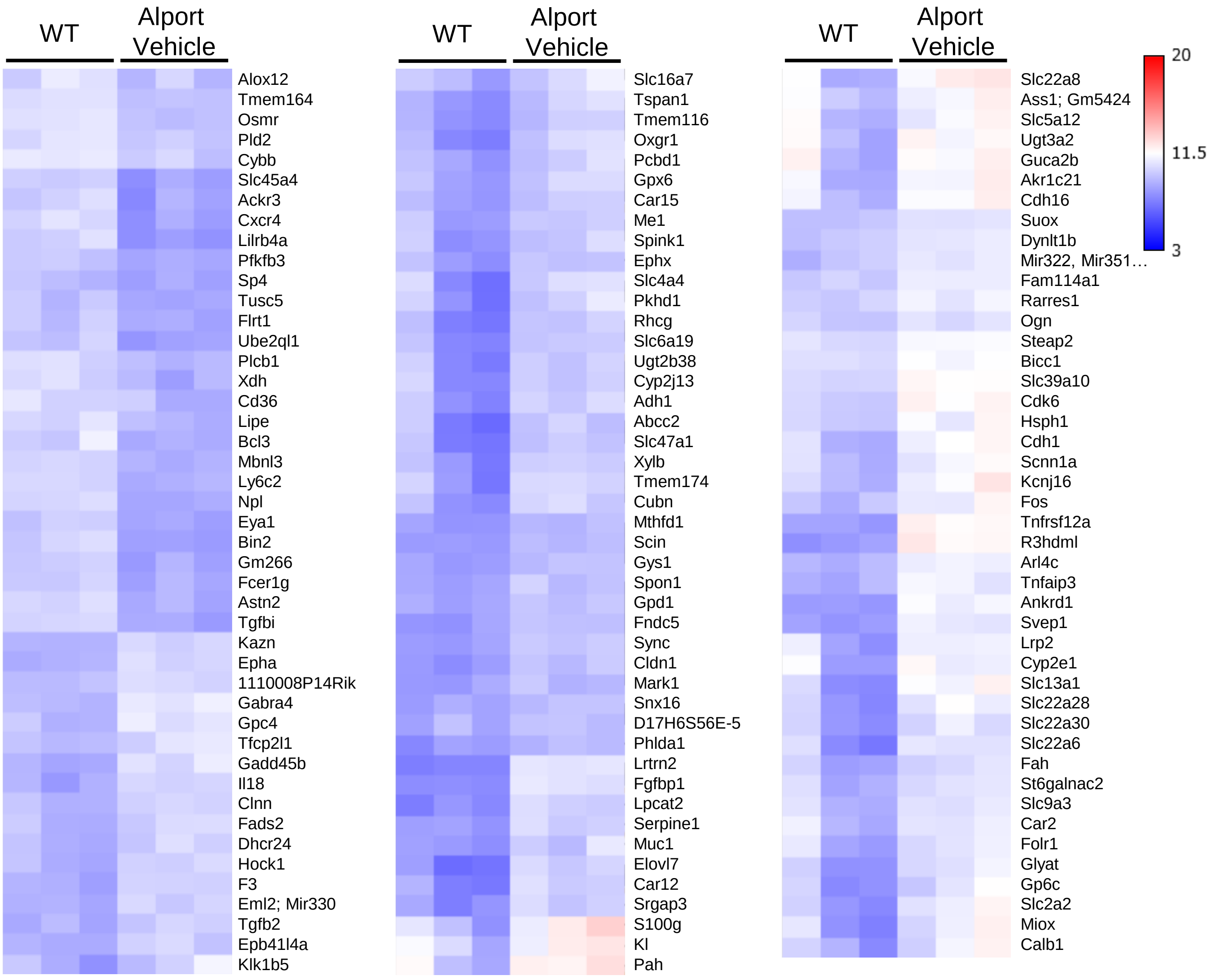

Supplementary Figure S6. Transcriptome analysis revealed the comprehensive effects of metformin on glomerular nephritis in glomerulus of *Col4a5* G5X-Alport syndrome mice
Heat map shows the fluctuated genes in the comparisons (WT vs Alport Vehicle).

Supplementary Figure S7

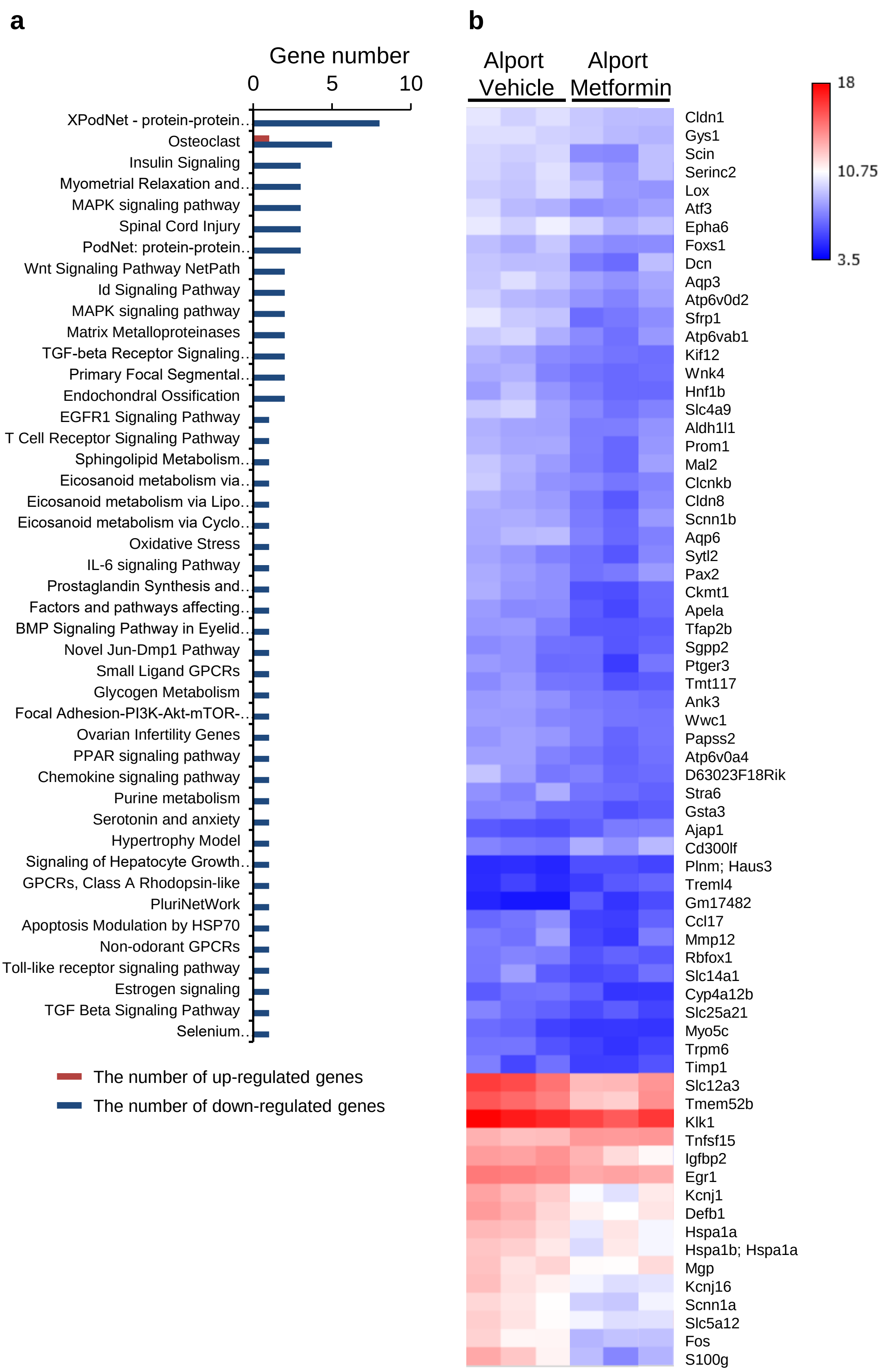

Supplementary Figure S7. Metformin-specific altered genes in the glomerulus of *Col4a5* G5X-Alport syndrome mice

(a) Pathway analysis of metformin-specific altered genes. (**b**) Heat map shows the altered genes in Alport metformin group compared with Alport vehicle (<-2, 2<, *P* value < 0.05) which did not change in Alport losartan group.

Supplementary Figure S8

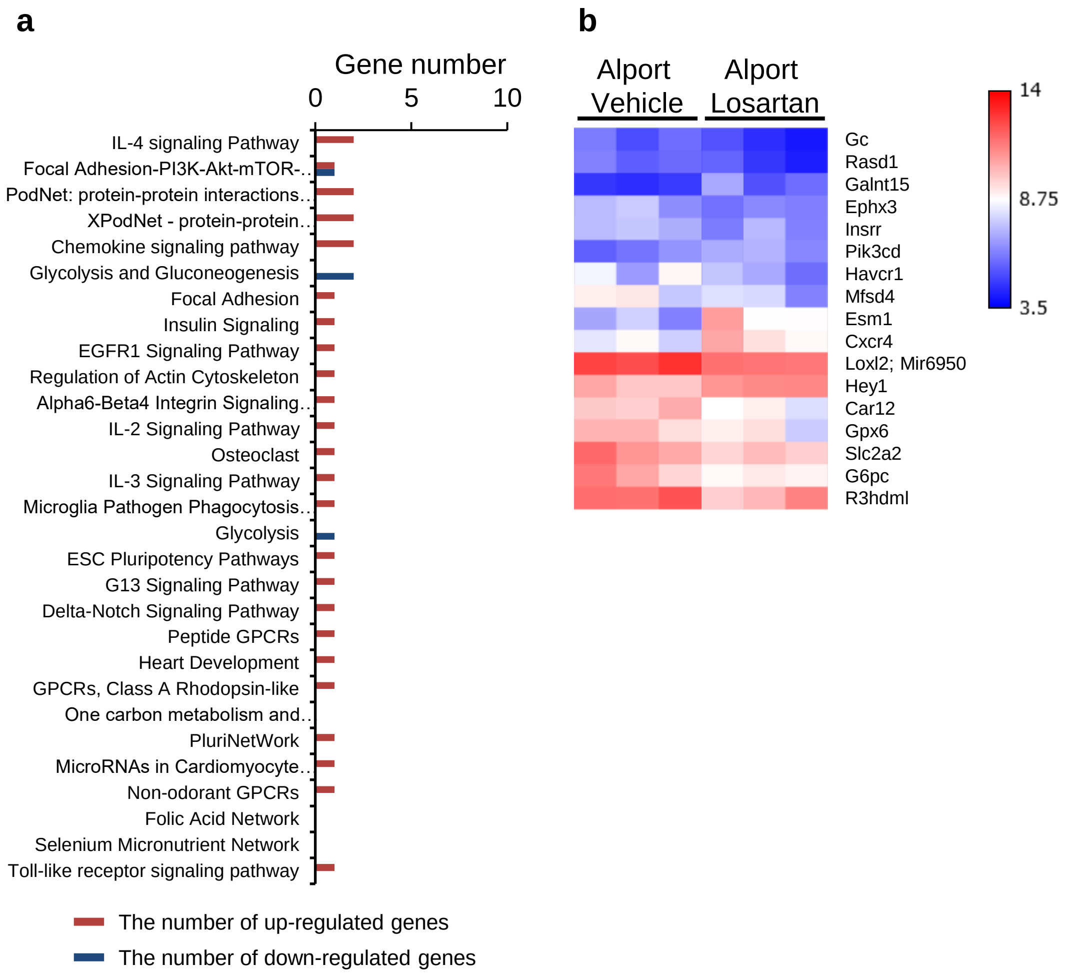

Supplementary Figure S8. Losartan-specific altered genes in the glomerulus of *Col4a5* G5X-Alport syndrome mice

(a) Pathway analysis of losartan-specific altered genes. **(b)** Heat map shows the altered genes in Alport losartan group compared with Alport vehicle (<-2, 2<, *P* value < 0.05) which did not change in Alport metformin group.

Supplementary Figure S9

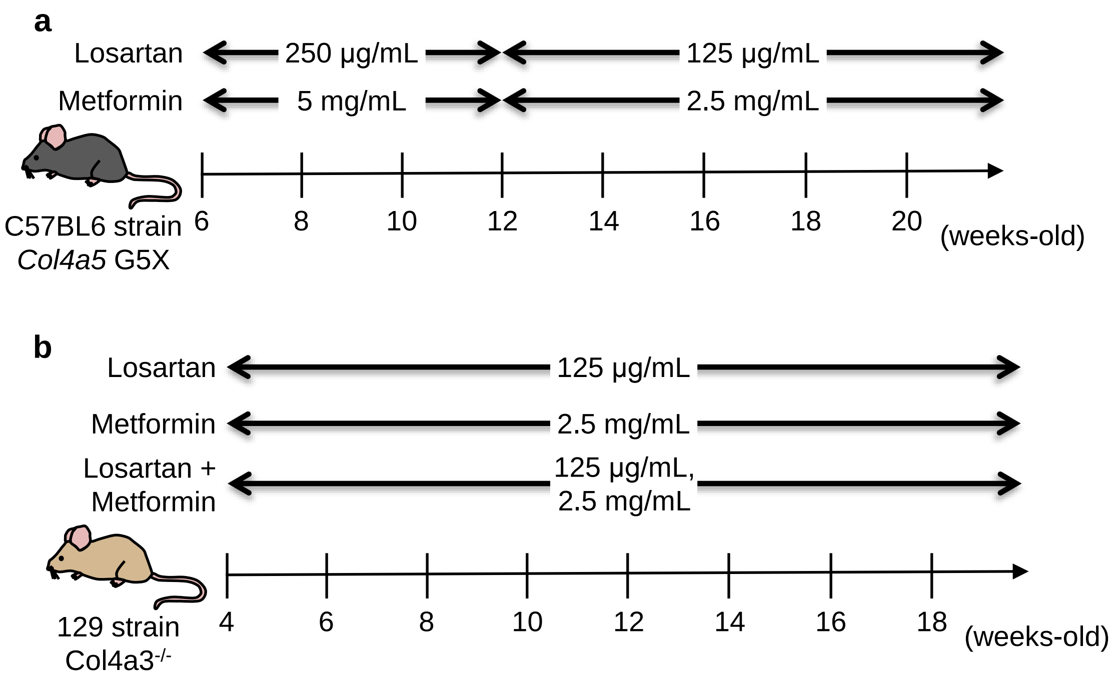

Supplementary Figure S9. Scheme of the treatments in mice for the survival study
(a) *Col4a5* G5X-Alport syndrome mice were treated with the indicated dose of losartan or metformin until all mice died. **(b)** *Col4a3*$^{-/-}$ Alport syndrome mice were treated with the indicated dose of losartan and/or metformin until all mice died. The images were drawn by S.K.

Supplementary Figure S10

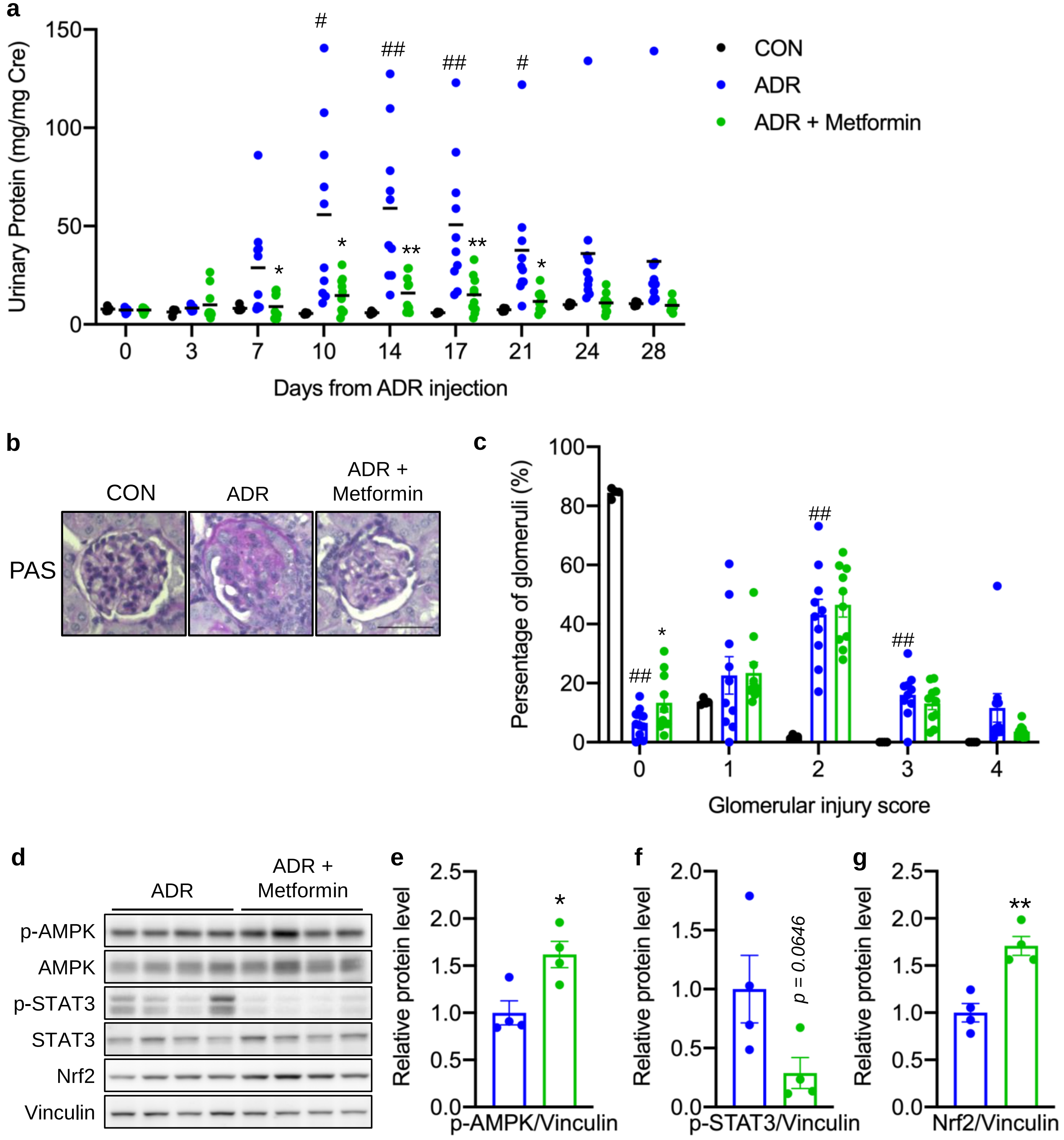

Supplementary Figure S10. Metformin protects against ADR-induced glomerulosclerosis
(**a**) Proteinuria score was calculated based on urinary protein and creatinine concentrations. Metformin reduced proteinuria in ADR nephropathy model. (**b**) Staining of renal sections was performed using PAS. Scale bar, 50 μm. (**c**) Glomerular injury scores were assigned based on the PAS-stained sections. Metformin ameliorated the severity of glomerulosclerosis in ADR nephropathy. Data are expressed as the means ± S.E. in control mice (n = 4), non-treated ADR, and metformin-treated ADR mice (n = 10 per group), *P* values were assessed by Dunnett's test ($^{\#}P < 0.05$, $^{\#\#}P < 0.01$ vs CON, $^{*}P < 0.05$, $^{**}P < 0.01$ vs ADR). (**d**) Whole kidney lysates were analyzed by immunoblotting. The full-length blots are presented in Supplementary Fig. S13. (**e-g**) The relative amount of proteins was quantified. Metformin increased the level of (**e**) phospho-AMPK and (**g**) Nrf2. Metformin decreased the level of (**f**) phospho-STAT3. Data are expressed as the means ± S.E. (n = 4 per group). *P* values were assessed by Student's *t* test ($^{*}P < 0.05$, $^{**}P < 0.01$ vs ADR).

Supplementary Figure S11

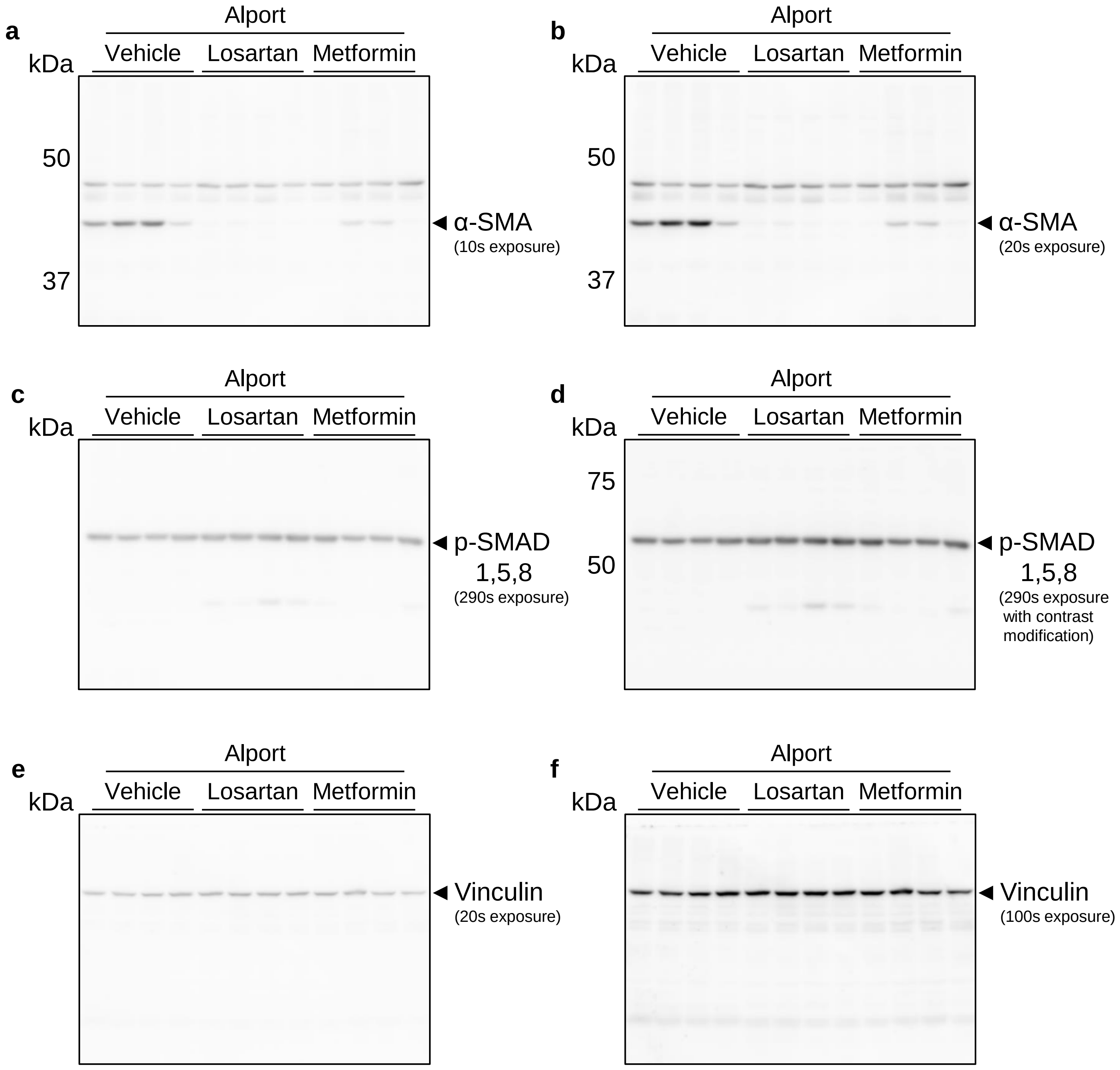

Supplementary Figure S11. Full length blots for Figure 2m

The full-length blots for Figure 2m with the indicated antibodies. Vinculin was used as loading control. Samples were derived from the same experiment, and gels/blots were processed in parallel.

Supplementary Figure S12

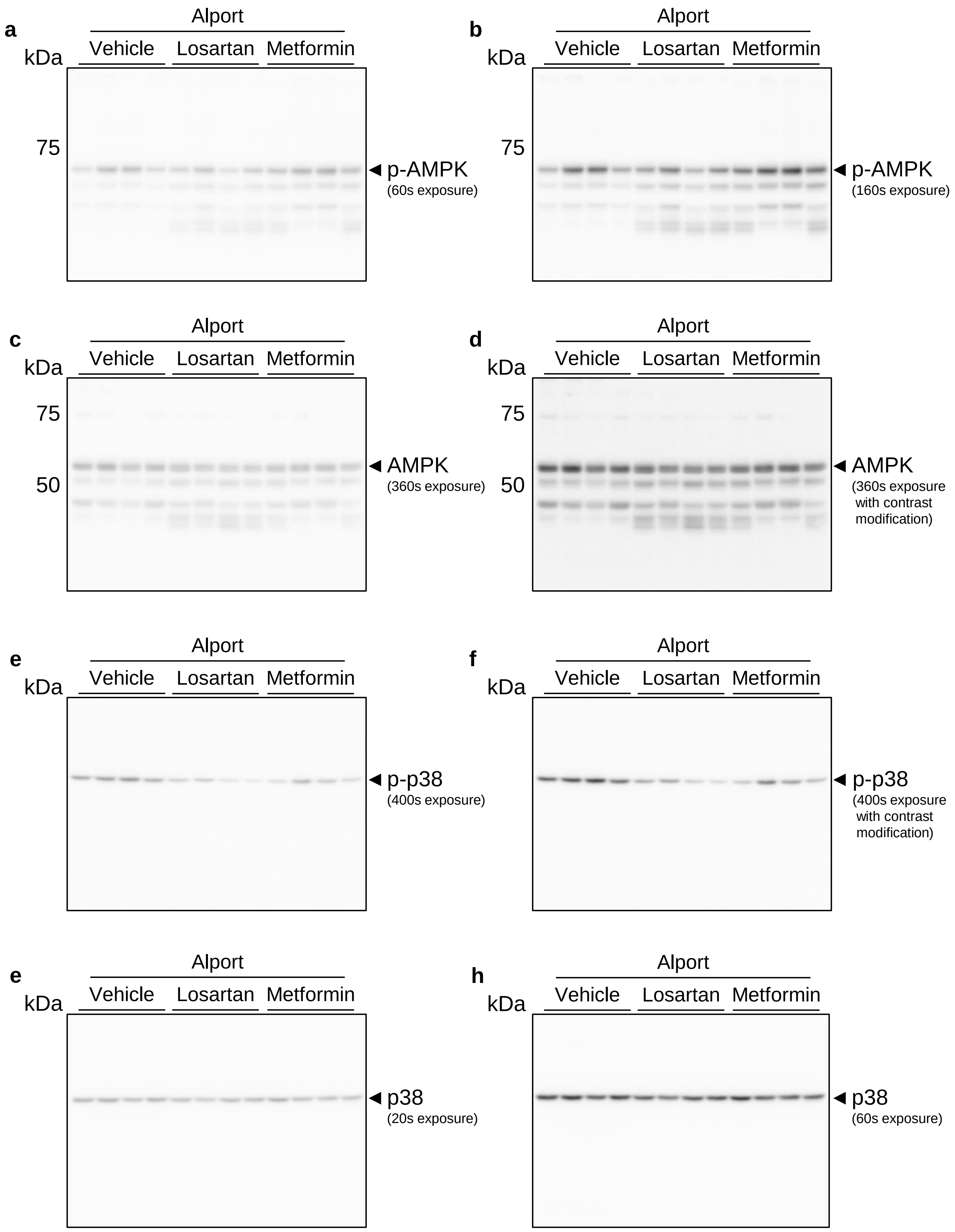

(Continue to the next page)

Supplementary Figure S12

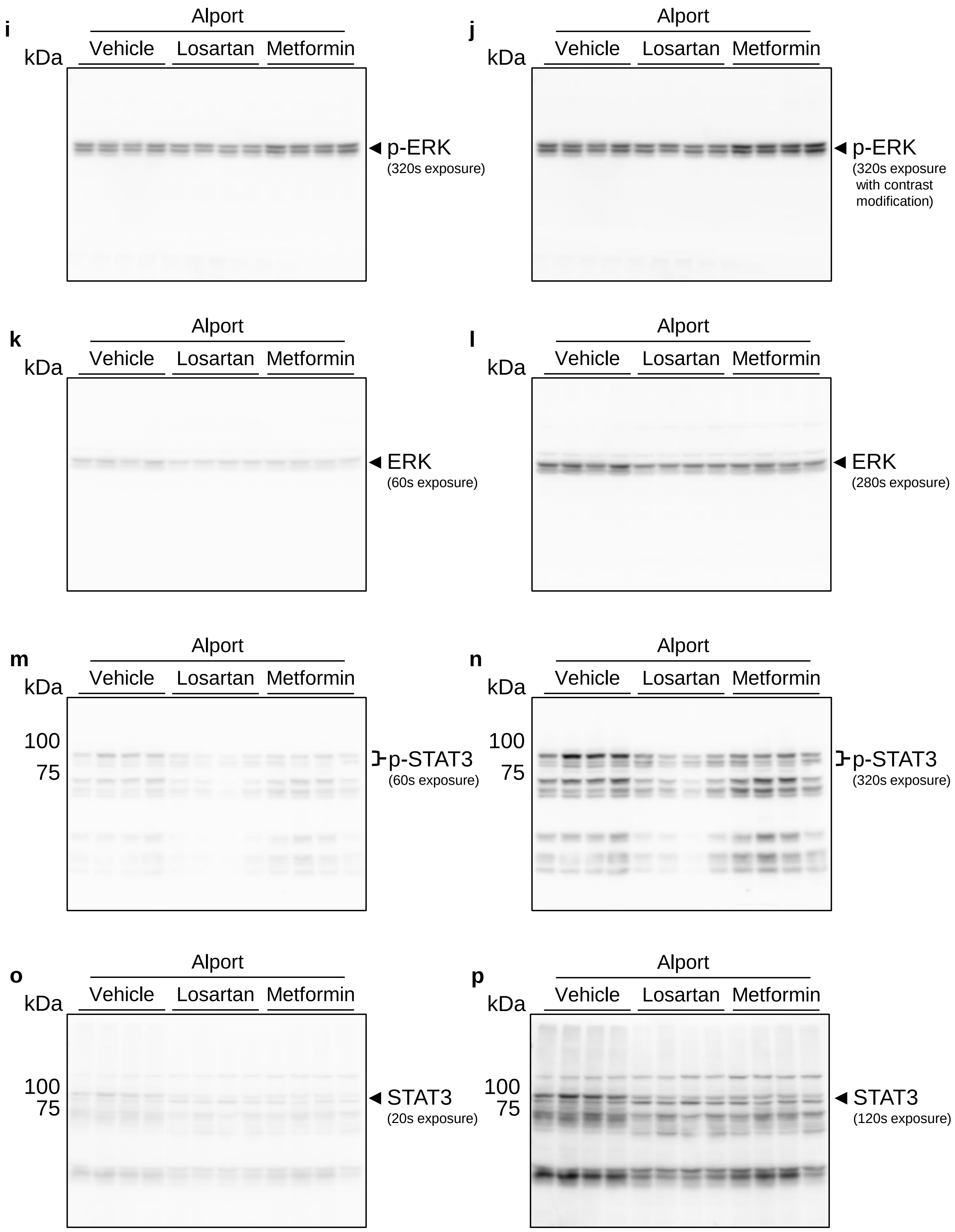

(Continue to the next page)

Supplementary Figure S12

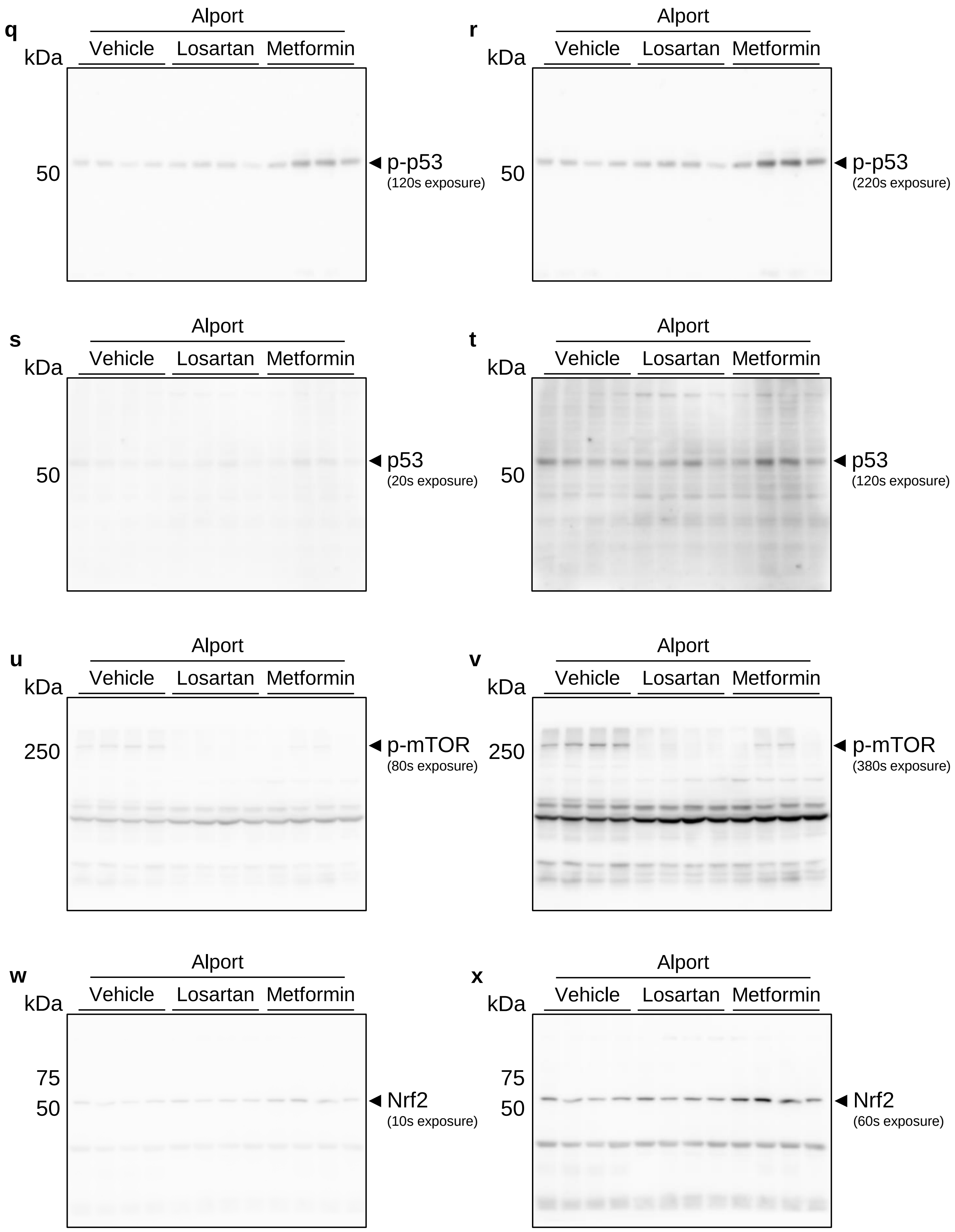

(Continue to the next page)

Supplementary Figure S12

y

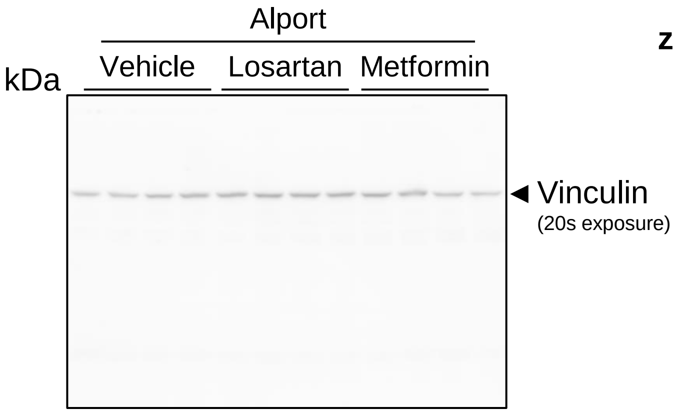

z

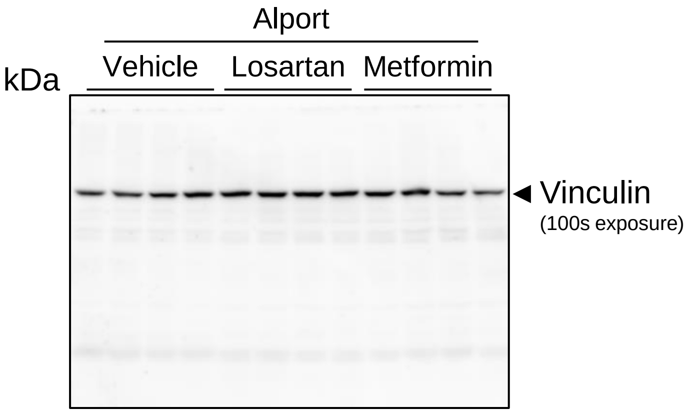

Figure S12. Full length blots for Figure 3a

The full-length blots for Figure 3a with the indicated antibodies. Vinculin was used as loading control. Samples were derived from the same experiment, and gels/blots were processed in parallel.

Supplementary Figure S13

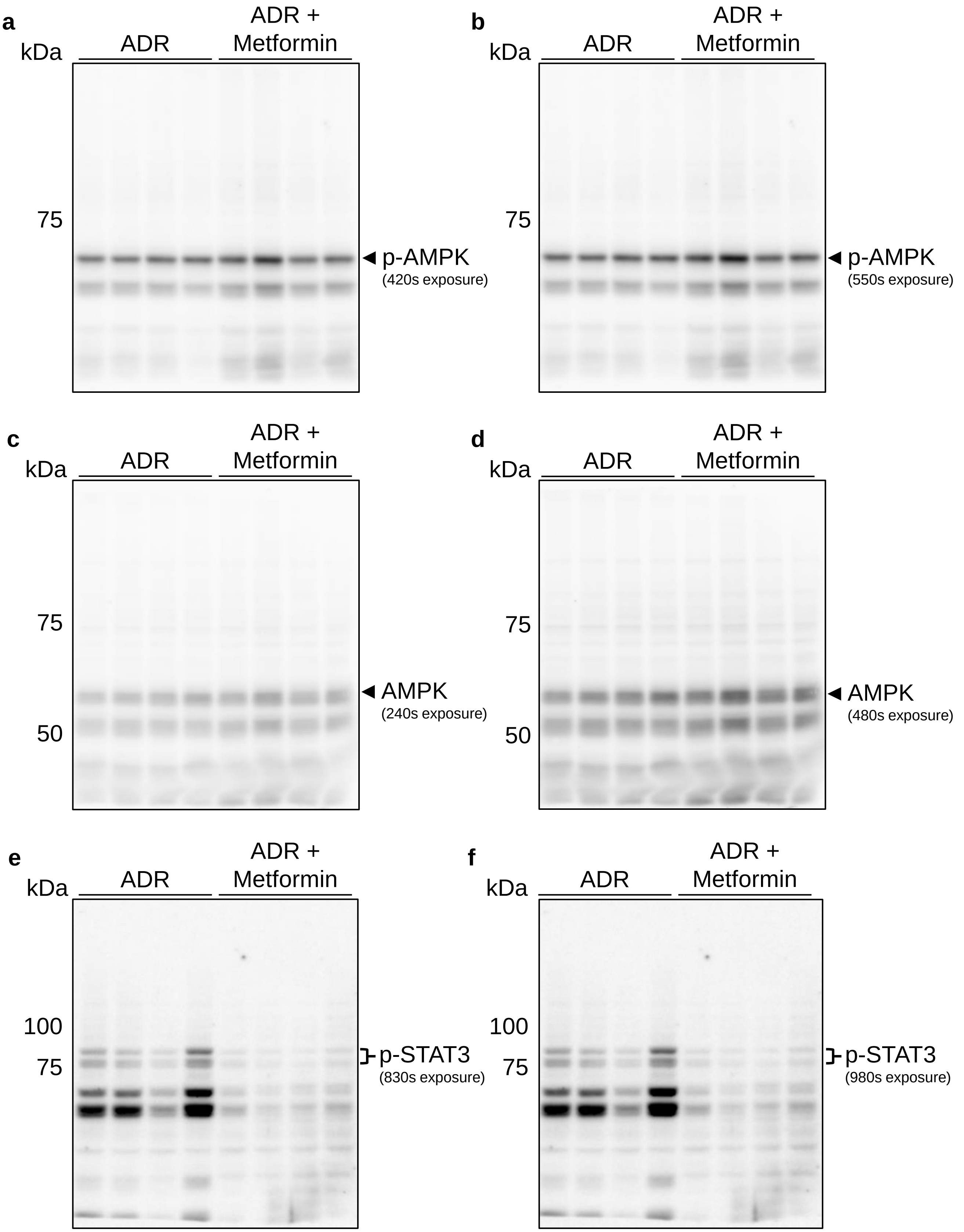

(Continue to the next page)

Supplementary Figure S13

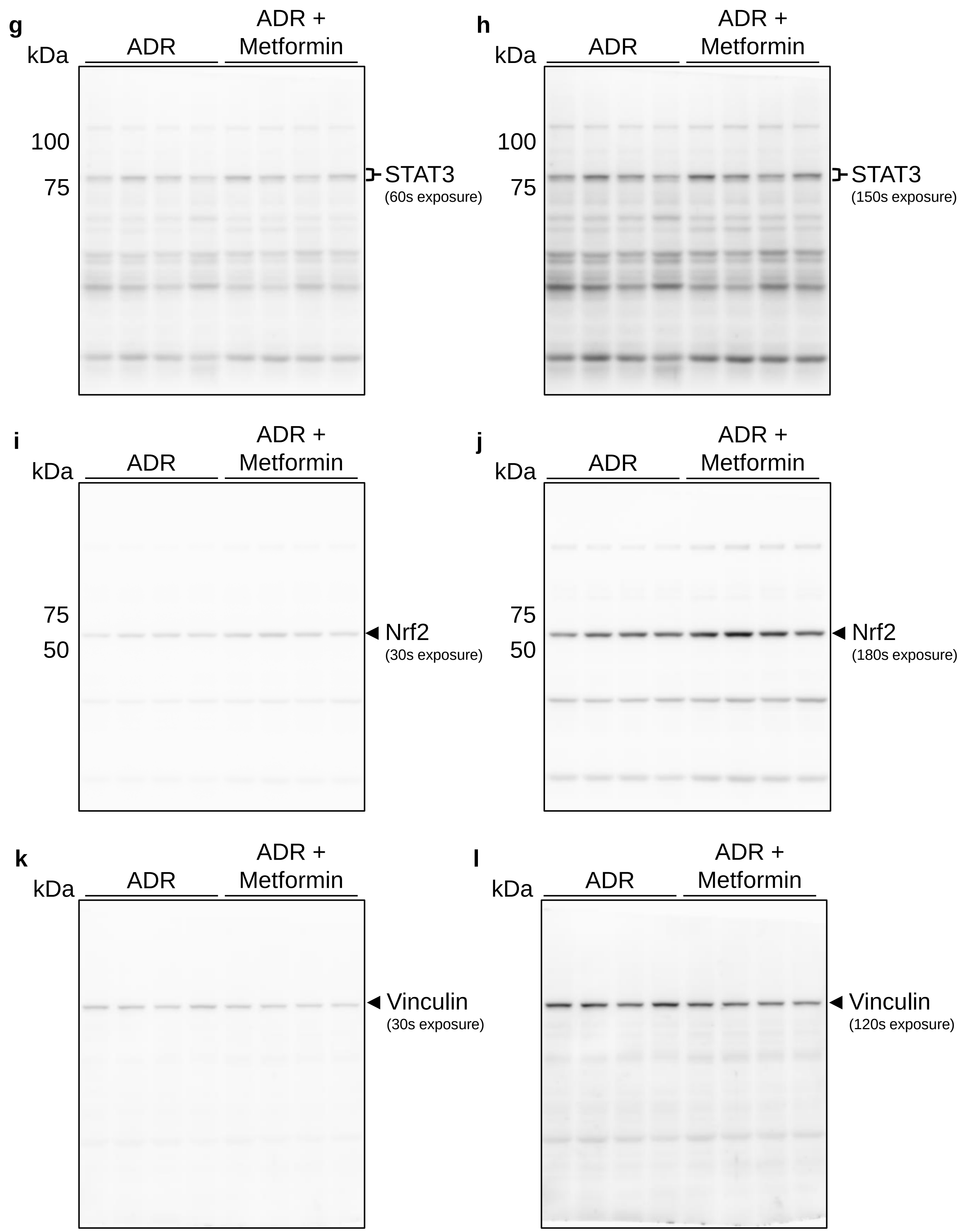

Supplementary Figure S13. Full length blots for Supplementary Figure S10d

The full-length blots for Supplementary Fig. 10d with the indicated antibodies. Vinculin was used as loading control. Samples were derived from the same experiment, and gels/blots were processed in parallel.
